# Mendelian Randomization Analysis Reveal the Role of Circulating Inflammatory Proteins in Mediating Functional Brain Networks and Peripheral Neuropathic Pain Effects

**DOI:** 10.1002/brb3.70751

**Published:** 2025-08-22

**Authors:** Wen‐Hui Liu, Hui‐Min Hu, Chen Li, Qing Shi, Yi‐Fan Li, Peng Mao, Bi‐Fa Fan

**Affiliations:** ^1^ Department of Pain Medicine China‐Japan Friendship Hospital Beijing China; ^2^ Graduate School Beijing University of Chinese Medicine Beijing China; ^3^ Department of Pain Medicine The Fourth Affiliated Hospital of Soochow University Suzhou China

**Keywords:** brain network functional connectivity, inflammatory factors, Mendelian randomization, neuropathic pain, resting‐state functional magnetic imaging

## Abstract

**Objective**: The perception of pain is thought to arise from the integration of information between multiple brain regions. Data from observational studies indicates that dysfunction of brain resting‐state functional networks is present in a wide range of peripheral neuropathic pain (pNP). The present study thus sought to investigate whether a causal relationship exists and to determine the potential mediating role of circulating inflammatory proteins in this association.

**Methods**: The resting‐state functional magnetic imaging phenotype is defined as a stable feature that quantifies the pattern of functional connectivity (i.e., synchronized activity) between different regions of the brain in the resting state of an individual. We gathered publicly available genome‐wide association study (GWAS) summary statistics for brain functional networks, including 191 rsfMRI phenotypes and postherpetic neuralgia (PHN) and trigeminal neuralgia (TN) in the FinnGen biobank. Furthermore, data were collected on genetic variation related to inflammation, including 91 circulating inflammatory proteins. We performed two‐sample MR analysis to investigate the causal effects of functional brain networks on PHN and TN. To explore the possible mediation of inflammatory factor changes between rsfMRI phenotypes and PHN and TN.

**Results**: The forward MR approach identifies five rs‐fMRI phenotypes that are causally associated with the risk of developing PHN. For instance, enhanced motor network connectivity was found to be associated with a reduced risk of PHN. Six rsfMRI phenotypes were identified as causally associated with TN risk. These brain network phenotypes mainly involve the default mode network (DMN), the sensory‐motor network (SMN), and the motor network, etc. Two‐step MR‐mediated analysis revealed that the inflammatory protein interleukin 20 receptor alpha (IL‐20RA) is a mediator of the pathway from the phenotype Pheno 12 of the brain motor network to PHN.

**Conclusion**: The findings provide valuable insights into potential targets for disease intervention and treatment at the level of functional brain networks.

## Graphical abstract

Motor network enhancement reduces postherpetic neuralgia (PHN) risk. lL‐20RA mediates of this brain network‐PHN effect, revealing a novel pathway for targeted interventions.

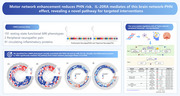

**Abbreviations**
ACC: anterior cingulate cortex
ADA: Adenosine deaminase
CEN: central executive network
CI: confidence interval
DMN: default mode network
fMRI: functional magnetic resonance imaging
GWAS: genome‐wide association study
IL‐20RA: interleukin 20 receptor alpha
IVs: instrumental variables
LD: linkage disequilibrium
LMTC: left medial temporal regions of cortex
MFG: middle frontal gyrus
mPFC: medial prefrontal cortex
MR: Mendelian randomization
NP: neuropathic pain
OR: odds ratio
PCC: posterior cingulate cortex
PCG: postcentral gyrus
PHN: postherpetic neuralgia
pNP: Peripheral neuropathic pain
rsfMRI: resting‐state functional magnetic imaging
SN: Salience network
SNPs: Single Nucleotide Polymorphisms
TN: trigeminal neuralgia

## 1 Introduction

Pain resulting from primary damage or dysfunction of the nervous system is defined as neuropathic pain (NP), which encompasses both central and peripheral. (Jensen et al. 2011; Baron et al. 2010) Peripheral neuropathic pain (pNP) is most often caused by peripheral nerve injury or dysfunction, and common disorders include postherpetic neuralgia (PHN) and trigeminal neuralgia (TN). TN is a chronic pNP condition that affects the face and jaw. It is characterized by periodic sharp electric shock‐like or knife‐like facial pain, which is usually triggered when the patient chews, washes and brushes his teeth, or touches his face. It is considered one of the most agonizing pains. (Araya et al. 2020; Maarbjerg et al. 2014) PHN is defined as pain that persists for more than three months following the resolution of shingles and represents one of the most prevalent forms of chronic pNP. (Scholz et al. 2019) As is the case with other chronic neurological disorders, patients with PHN and TN display a range of signs indicative of peripheral and central neuropathy, as well as corresponding changes in the brain regions involved, including nociceptive sensitization, abnormal pain, and loss of sensation. (Oaklander 2001; Dworkin 2002; Baliki et al. 2008) The pathogenesis of NP is complex and includes anatomical and structural alterations and functional impairments that can lead to peripheral sensitization, central sensitization, dysfunction of the descending inhibitory system, inflammatory responses, and abnormal alterations in ion channels. (Cohen and Mao 2014; Loeser and Treede 2008; Nieto‐Rostro et al. 2018) Among these is central sensitization, which represents an important pathogenic mechanism of NP. The maintenance of NP is primarily dependent on the presence of central sensitization. It is frequently accompanied by symptoms such as anxiety and depression, and in severe cases, it can even lead to self‐harm and suicide, which represents a significant challenge that requires immediate attention. (Bendtsen et al. 2020).

Pain is defined as a perceptual and emotional experience that involves high‐level integration of the central nervous system. The generation of pain relies on the transmission of peripheral signals to the brain and the synergistic processing of multiple brain regions. The presence of chronic NP has been demonstrated to be associated with dysfunction in key brain networks. These networks are involved in self‐monitoring, pain processing, and salience detection. In previous studies, researchers have identified a disruption in synchronization and connectivity in areas of the brain involved in self‐monitoring, pain processing, and common areas of salience detection in individuals with chronic NP. (Cauda et al. 2010; Seifert and Maihöfner 2009) Neuroimaging studies are currently being employed to identify pain‐modulating systems. Functional magnetic resonance imaging (fMRI), which is based on blood oxygenation level‐dependent imaging, is used to detect neural activity by measuring changes in deoxyhemoglobin, a paramagnetic substance in the blood. This technique has a wide range of applications. (Finn et al. 2015; Nemirovsky et al. 2023; van den Heuvel and Hulshoff Pol 2010) The existence of multiple brain‐intrinsic resting state networks (RSNs) has been identified using fMRI, including the salience network (SN), motor network, default mode network (DMN), and central executive network (CEN). These networks interact dynamically to collectively modulate pain perception. Studies on chronic NP have found that pain is associated with alterations in the functional connectivity of the anterior cingulate cortex (ACC) with the sensory‐motor cortex and between the striatum and thalamus. SN has been demonstrated to be a significant factor in the maintenance of attentional resources and the amplification of pain perception. Enhanced connectivity within the DMN has been shown to promote the persistence of pain, often manifesting as excessive self‐preoccupation. Furthermore, diminished function of the CEN has been linked to a diminished ability to inhibit pain. This imbalance in the dynamics of the triple network constitutes a central feature of central sensitization in chronic pain. (Gopinath et al. 2011; Weizman et al. 2018; Alhajri et al. 2023; Wu et al. 2020) The brain's regional characteristics and the brain's inter‐regional structural connections can be employed to identify differences in brain structure between pain disorders. (Qiu et al. 2021) In recent years, randomized controlled studies have demonstrated that intra‐DMN connectivity, including left medial temporal regions of cortex (LMTC)‐middle frontal gyrus (MFG) and LMTC‐posterior cingulate cortex (PCC) connectivity, is significantly enhanced in patients with PHN. Consistent with hyperactive endoreceptor processing. In contrast, inter‐hemispheric connectivity tends to be reduced between the hemispheres. Patients with TN develop abnormalities in SN‐related areas such as significant activation of the right postcentral gyrus (PCG) and significant inhibition of the left supplementary motor area. These studies provide more evidence for the phenotypic quantification of pain network pathology by rsfMRI. (Kim et al. 2023; Liu et al. 2013; Wang et al. 2024).

Furthermore, the inflammatory response may result in the production of NPs. A number of studies have demonstrated that inflammatory cell infiltration can result in inflammatory responses, hemorrhagic necrosis, neuronal loss, and demyelination in peripheral nerves, which in turn can induce changes in nerve remodeling and promote peripheral sensitization. (Ji et al. 2016; Sommer et al. 2018) There is a close relationship between inflammatory molecules and fMRI. (Williams et al. 2022) This suggests that inflammatory proteins may mediate the association between brain network function and NP development.

Because of the potential confounding bias that may exist in observational studies, it is not clear what the causal relationship is between brain network function and PNP, and whether inflammatory factors are involved in mediating the association. In order to study the effect of brain network function on pNP, we employed a Mendelian randomization (MR) research methodology that utilizes genetic variants as proxy instrumental variables (IVs) for exposure factors to infer the causal relationship between exposure factors and disease. This methodology effectively avoids confounding factors and reverse causation by comparing the differences in disease incidence between individuals with different genetic variants. (Davies et al. 2018; Emdin et al. 2017) In this study, we initially sought to ascertain the causal effects of brain network function on two prevalent pNP types. To this end, we employed two‐sample MR analysis, utilizing recent GWAS summary statistics on fMRI. Subsequently, the causal effects of inflammatory factors on pNP were investigated. Finally, we examined the mediating role of inflammatory factors on brain network function‐pNP using two‐step MR mediation analysis.

## 2 Materials and Methods

### 2.1 Study Design

**FIGURE 1:**
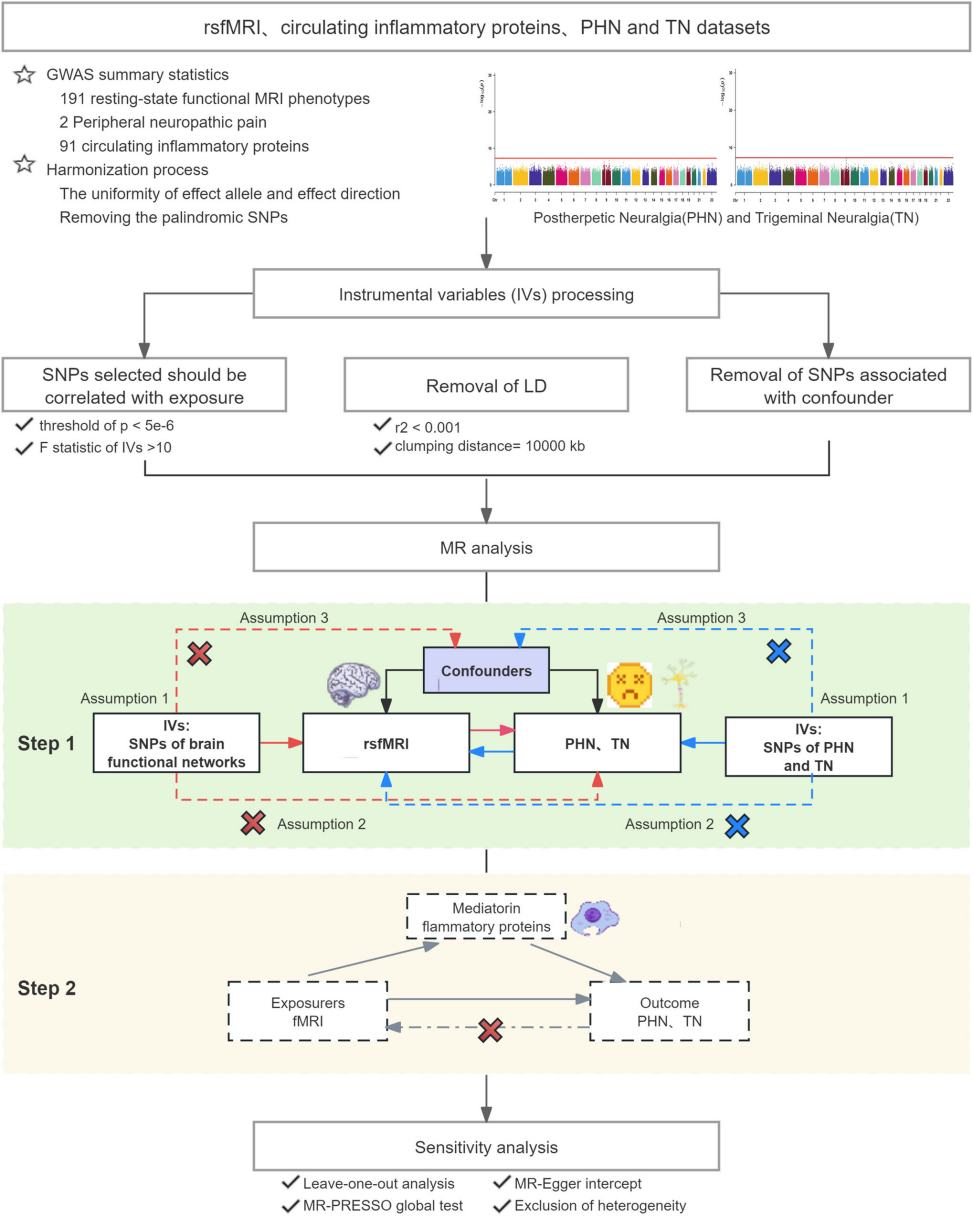
Workflow for bidirectional MR analysis and inflammatory protein mediation analysis between brain rsfMRI and PHN and TN. Briefly, SNPs that were independent and significantly associated with exposure were selected as IVs, and SNPs associated with confounders were excluded. After rigorous quality control of the IVs, Step 1 was performed for MR analysis to infer causal relationships between rsfMRI phenotypes and PHN and TN. Step 2 MR analysis was performed to infer the causal relationship between inflammatory proteins and PHN and TN and inflammatory protein‐mediated effects. Finally, sensitivity analysis was used to assess the robustness of MR inferences.

This study used a two‐sample MR analysis, based on genome‐wide association study (GWAS) summary statistics, to examine the genetic causal effects of exposure on outcomes. The study was conducted in accordance with the three fundamental assumptions of the MR analysis: (1) the correlation assumption: the IVs were strongly associated with exposure; (2) the exclusivity assumption, which indicated that the IVs exerted an influence on the outcome exclusively through the target exposure pathway; and (3) the independence assumption, which demonstrated that the IVs were not confounded by potential confounding factors. As shown in Figure 1, in Step 1, we assessed the causal effects of brain function networks on two common types of pNP and performed a bidirectional analysis, where brain functional networks with positive effects should not have reverse causality in mediation analysis. In Step 2, we analyze the causal effects of 91 circulating inflammatory proteins on two common pNPs, as well as the analysis of inflammatory factors mediating the pathway from brain functional networks to pNPs. In accordance with the ethical standards governing research, the requisite ethical clearance was not required, as all data utilized in this study were publicly accessible.

### 2.2 Sample and Data Sources

Although fMRI metrics associated with triple networks have been found to play an important role in the pain process in previous studies, the findings are highly variable and involve numerous brain networks. Consequently, the MR study utilized data from 191 fMRI phenotypes. The rsfMRI phenotypes data utilized in this study were derived from the most comprehensive meta‐analysis to date, published by Zhao et al. (2022). This analysis collected individual‐level rsfMRI data from four independent studies, including the UKB, Adolescent Brain Cognitive Development, Philadelphia Neurodevelopmental Cohort, and Human Connectome Project, which included multiple cohorts. There were 34,691 samples of British people from the UK Biobank. (Elliott et al. 2018; Grasby et al. 2020; Zhao et al. 2019) By following a unified UKB brain imaging pipeline to coordinate rsfMRI processing, spatially independent component analysis was performed to characterize functional brain regions and corresponding functional connectivity in UKB, ultimately generating 1,777 neuroimaging phenotypes that were used to explore the genetic architecture of intrinsic brain activity. From the 1777 traits screened for phenotypes significantly affected by genetic variation (5×10‐8), the GWAS selected a total of 191 phenotypes, including SN, DMN, CEN, somatomotor network, attentional network, limbic system, and visual network, including 75 amplitude traits (nodes) reflecting spontaneous neural activity in the region, 111 pairwise functional connectivity, and 5 global functional connectivity. Amplitude features reflect changes in rsfMRI signals and are used to assess the intensity of neural activity in relevant functional areas.

Furthermore, updated GWAS summary statistics for two prevalent pNP types were obtained from the FinnGen Biobank Round 11 analysis, with GWAS IDs for finngen_R11_G6_POSTZOST and finngen_R11_G6_TRINEU, respectively, and diseases for PHN (36,089 individuals, case 420, control 395,832) and TP (397,805 individuals, case 1973, control 395,832).The present study utilized GWAS pooled data for 91 circulating inflammatory proteins, which were employed for mediation analyses. These data were drawn from the most comprehensive meta‐analysis published by Zhao J. H. et al. in 2023, with a cumulative participant count of up to 14,823 individuals. All participants in the inflammatory gene variant data were of European ancestry.

### 2.3 Selection and Harmonization of Genetic IVs

The selected IVs must satisfy the three assumptions of the MR analysis in order to guarantee the robustness and reliability of the MR analysis. (1) Relevance: To meet strong associations between IVs and exposures, we selected the genetic IVs with a genome‐wide significance threshold of *p* < 5 × 10^− 6^. Furthermore, in order to ensure a robust association between IVs and exposure, we calculated the F‐statistic for IVs. Any single‐nucleotide polymorphisms (SNPs) with an *F* value (*β*2 / se2) less than 10 were considered weakly biased instrumental variables and wetr excluded from the analysis. (Burgess et al. 2017) (2) independence: The linkage disequilibrium (LD) between SNPs was then removed, as strong LD leads to bias (*r^2^* < 0.001, clumping distance = 10,000 kb). (3): Exclusivity: we used PhenoScanner data to eliminate potential confounders. (Kamat et al. 2019) Prior to MR analysis, the exposure and resultant data were harmonized using the TwoSampleMR (v.0.5.6) R packag(https://mrcieu.github.io/TwoSampleMR) in the R software (R.4.3.1), and palindromic SNPs with a minimum allele frequency MAF close to 50% were removed.

### 2.4 Statistical and Sensitivity Analysis

Two‐sample MR analyses were conducted using the TwoSampleMR R package. The primary analytical method employed was multiplicative random‐effects inverse variance‐weighted (IVW), which was utilized to assess the causal effect of exposure on outcomes. The four MR Egger, weighted median, simple model, and weighted model are auxiliary methods. The significant threshold was set as two‐tailed *p* < 0.05 in IVW (Zuber et al. 2022).

The study utilized the FDR correction method. In order to test for potential horizontal pleiotropy effects and to reduce bias in MR estimation, we employed the *p*‐value of the MR‐PRESSO Global test and the *p*‐value of the intercept obtained from the MR Egger regression. Both Cochran's *Q* statistic and Rucker's *Q* statistic were employed to identify heterogeneity in the MR analysis. A value of *p* > 0.05 indicates the absence of heterogeneity in the IVs. A leave‐one‐out sensitivity analysis was employed to assess the influence of individual SNPs on the outcomes of the analysis. (Verbanck et al. 2018; Cohen et al. 2015; Burgess and Thompson 2017; Bowden and Holmes 2019) Causality was considered significant if the following three conditions were met: (1) The IVW *p*‐value was less than 0.05. (2) There were no potential horizontal pleiotropy effects or heterogeneity. (3) The estimates from the IVW and MR‐Egger methods were in the same direction.

### 2.5 Mediation Analysis

The objective of this study was to investigate the mediating pathways from functional brain networks to associated pNP through the analysis of circulating inflammatory proteins. Brain functional networks and circulating inflammatory proteins that had a significant causal effect on the associated pNPs were included in the mediation analysis by two‐sample analysis. We estimated the causal effect of 191 fMRI phenotypes on circulating inflammatory proteins. Finally, we quantified the indirect effects of fMRI phenotypes on PHN and TP by measuring the concentration of circulating inflammatory proteins. The indirect effects and their standard errors were assessed using the “product of coefficients” and “delta” methods, respectively. (Yao et al. 2022).

## 3 Results

### 3.1 MR of rsfMRI Traits on PHN and TN

**FIGURE 2:**
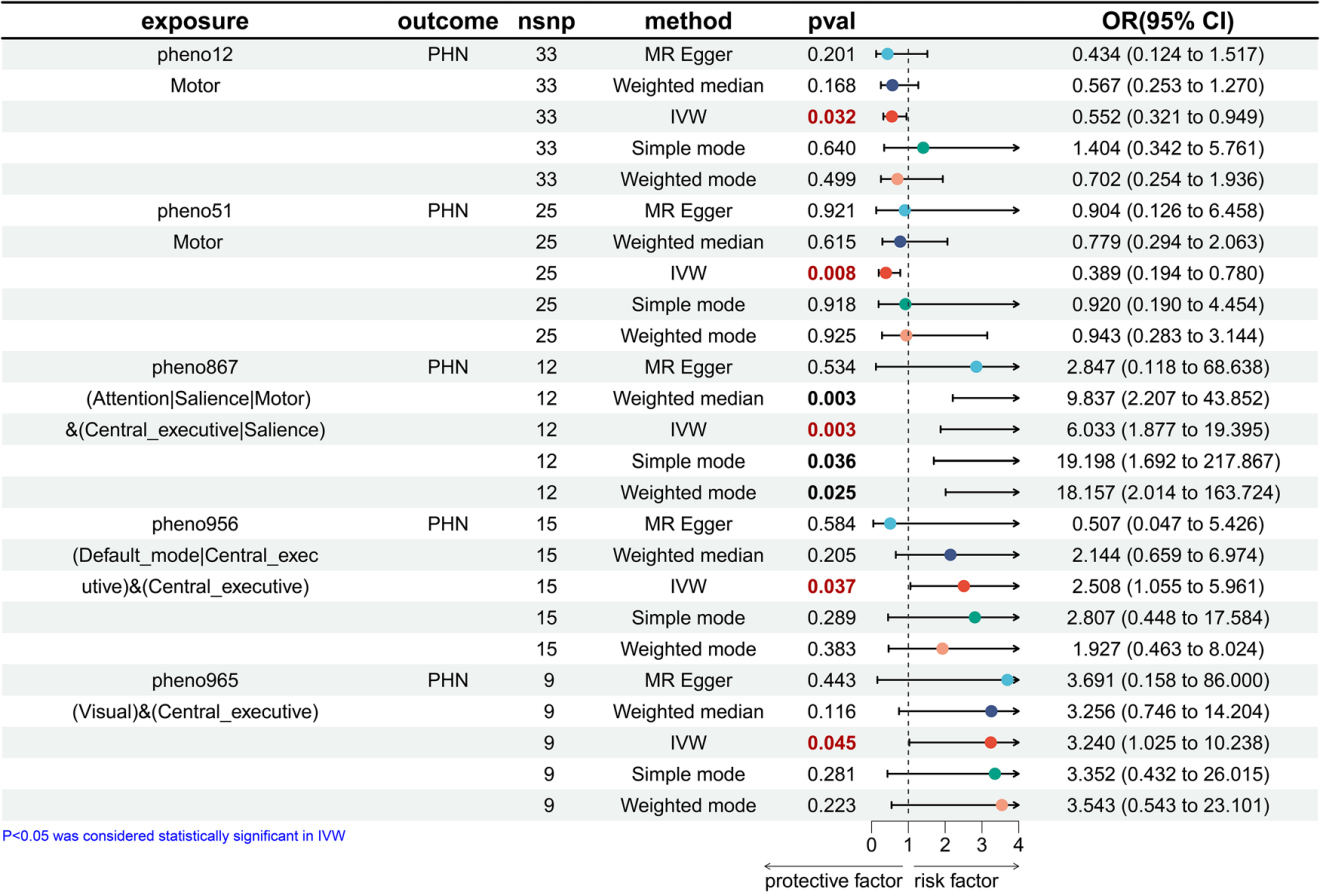
Mendelian randomization results of causal effects between brain rsfMRI phenotypes and PHN.

**FIGURE 3:**
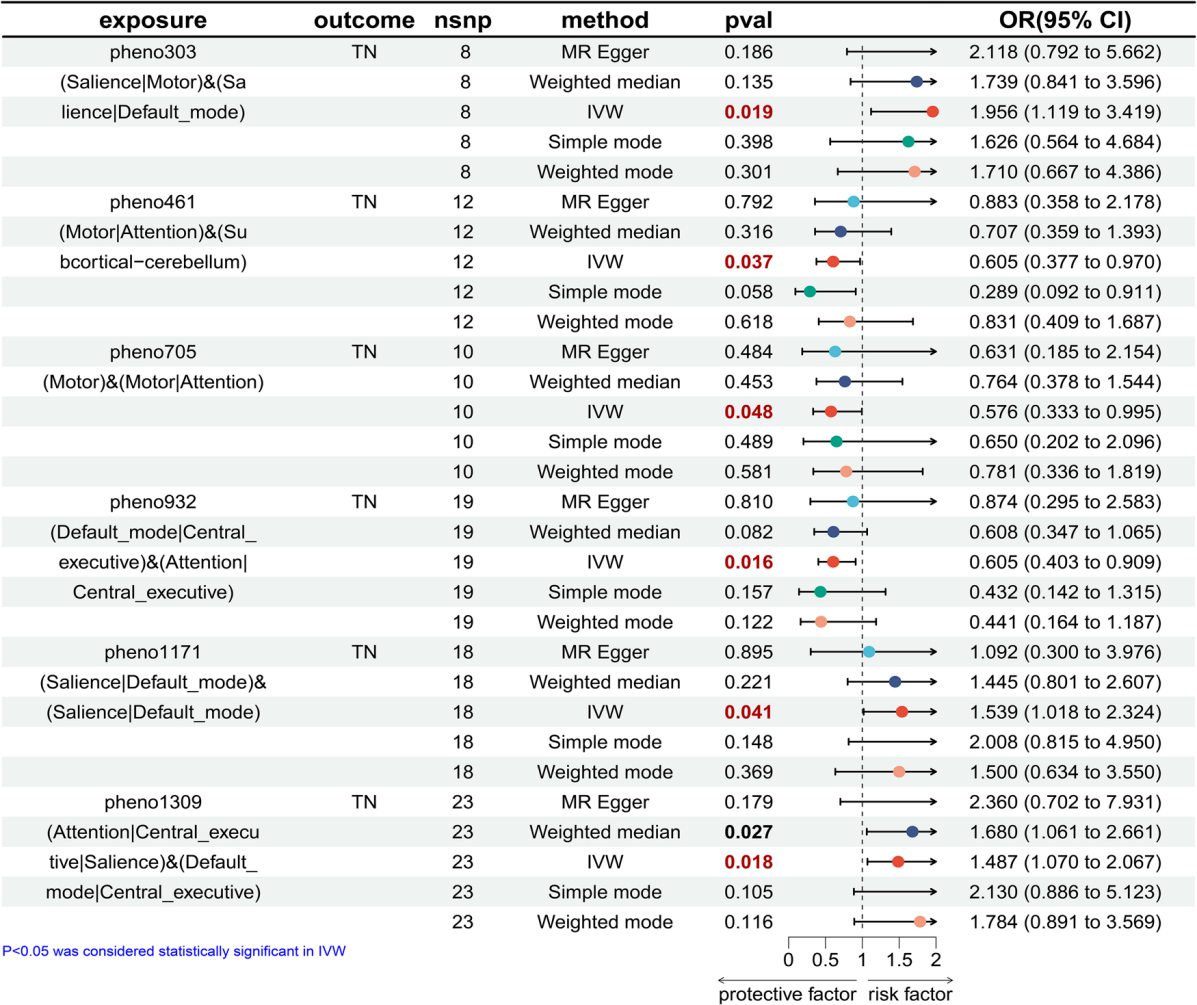
Mendelian randomization results of causal effects between brain rsfMRI phenotypes and TN.

**FIGURE 4:**
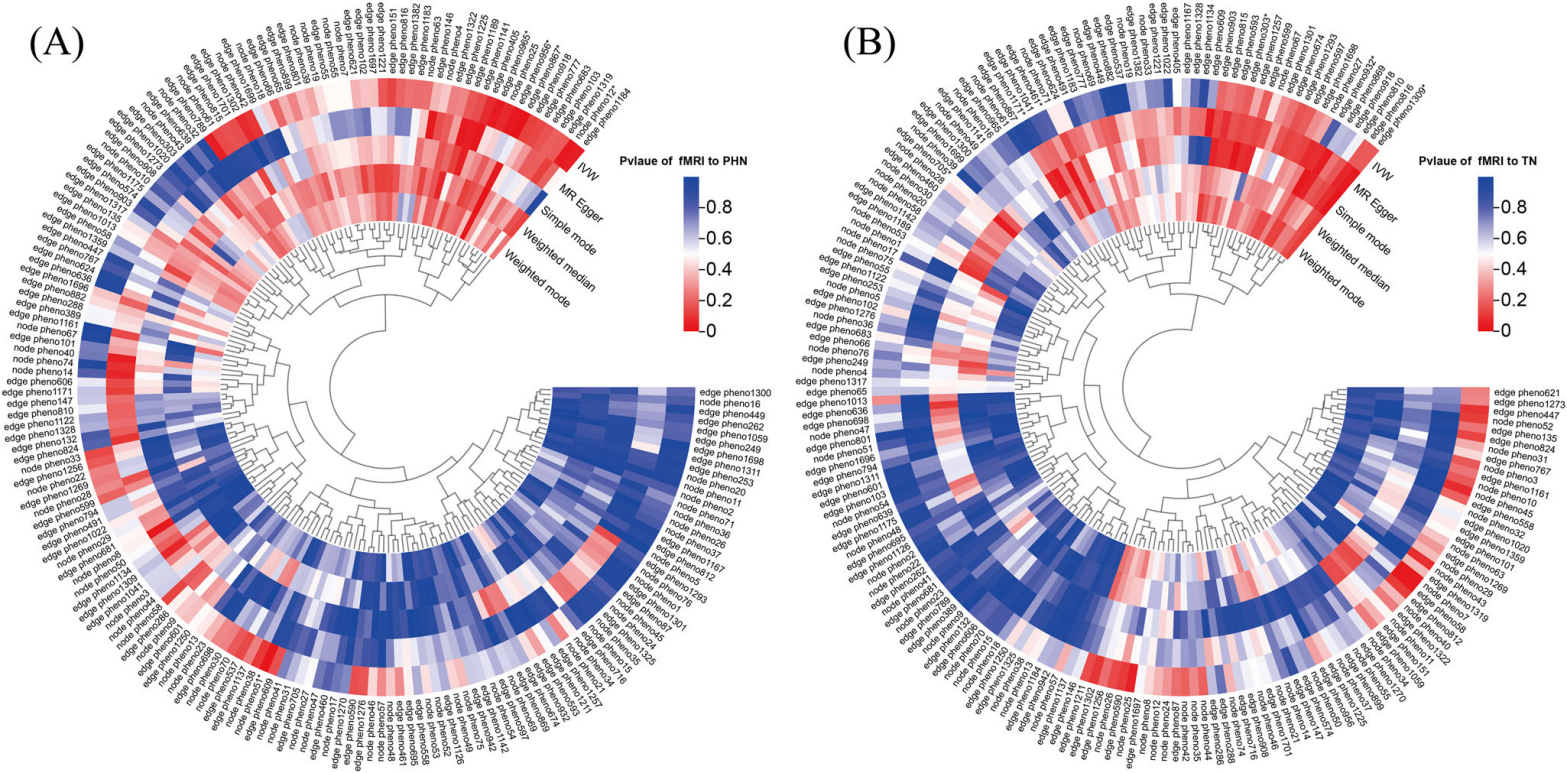
The rightmost side of the heatmap shows a dendrogram based on Pval values between 0 and 1, with colours fading from red to blue, with redder heatmap colours indicating stronger causality. The outer part of the circular heat map shows the GWAS number of the data, and positive results are marked with an *. The outermost circle of the heat map is the main research method IVW for our inclusion criteria, the five methods we labelled in the opening of the circle heat map. (A) *p*‐value of fMRI to PHN, (B) *p*‐value of fMRI to TN.

In forward MR analysis, we identified 11 functional brain networks (rsfMRI phenotypes) that may be causally related to PHN and TN under investigation. (Figures 2 and 3), and we also visualized heatmaps for the forward analysis (Figure 4). None of the 11 phenotypes had reverse causal effects (Supplementary Material 1: Figure S1). See Supplementary Information for location and rsfMRI network for corresponding phenotypes. (**Supplementary Material 2**: Table S1).

### 3.2 Effects of Brain Functional Networks on PHN

In this study, we identified five rsfMRI phenotypes that were causally associated with PHN. The five phenotypes are related to the activity of the paracentral or postcentral, paracentral, paracentral or frontal or supplementary motor area and frontal, precuneus or angular or cingulate and frontal, and cuneus or occipital and frontal regions, and mainly affect motor network, attention network or SN or motor network‐CEN or SN, DMN or CEN and CEN, and visual and CEN.

As shown in Figure 2. In the motor network, the risk of developing PHN is reduced by 44.8% for every 1 s.d. increase in amplitude traits (nodes) in the paracentral or postcentral region (IVW: odds ratio (OR) = 0.552, 95% confidence interval (CI): 0.321–0.949, *p* = 0.032), and the risk of developing PHN is reduced by 61.1% for every 1 s.d. increase in amplitude traits (nodes) in the paracentral region (IVW: OR = 0.389, 95% CI: 0.194–0.780, *p* = 0.008). Activity in the paracentral or frontal or supplementary motor area and frontal regions was positively associated with PHN risk. Activity in these brain regions influenced functional connectivity in the multimeshed network (attention network or SN or motor network and CEN or SN), and an s.d. increase in functional connectivity of these networks was associated with an upper risk of PHN (IVW OR = 6.033, 95% CI: 1.877–19.395, *p* = 0.003).

Neural activity in the precuneus or angular or cingulate and frontal lobes at rest was positively correlated with PHN, with a 150.8% increase in the risk of developing PHN for every 1 s.d. increase in functional connectivity of the DMN or CEN and CEN (IVW OR = 2.508, 95% CI: 1.055–5.961, *p* = 0.037). The functional connectivity between the cuneus or occipital and frontal regions was also positively associated with the risk of PHN. An s.d. increase in the functional connectivity between the visual and CEN increased PHN risk (IVW OR = 3.240, 95% CI: 1.025–10.238, *p* = 0.045).

### 3.3 Effects of Brain Functional Networks on TP

As shown in Figure 3. In this study, we identified six rsfMRI phenotypes that were causally associated with TN. The six phenotypes are related to the activity of the rolandic operculum or supramarginal gyrus or insula and insula or cingulate gyrus, postcentral or precentral and cerebellum, paracentral and postcentral or precentral, precuneus or cuneus or cingulate and precuneus or supplementary parietal, insula or cingulate and frontal, and parietal and temporal regions and mainly affect functional connectivity of SN or motor network and SN or DMN, motor network or attention network and subcortical‐cerebellum, motor network and motor network or Attention network, DMN or CEN and Attention network or CEN, SN or DMN and SN or DMN, and attention network or CEN or SN and DMN or CEN.

The functional connectivity between postcentral or precentral and cerebellum regions was positively associated with the risk of TN, and 1 s.d. increase in the functional connectivity between the SN or motor network and SN or DMN increased TN risk by 95.6% (IVW OR = 1.956, 95% CI: 1.119–3.419, *p* = 0.019). Functional connectivity between the rolandic operculum or supramarginal gyrus or insula and insula or cingulate regions was negatively correlated with the risk of TN, and 1 s.d. increase in functional connectivity between the motor network or attention network and subcortical‐cerebellum network reduced the risk of TN by 39.5% (IVW OR = 0.605, 95% CI: 0.377–0.970, *p* = 0.037). Functional connectivity between the paracentral and postcentral or precentral regions was negatively correlated with the risk of TN, and 1 s.d. increase in functional connectivity between the motor and motor or attention network, the risk of TN was reduced by 42.4% (IVW OR = 0.576, 95% CI: 0.333–0.995, *p* = 0.048). Functional connectivity between the insula or cingulate and frontal regions was also negatively correlated with the risk of TN, and 1 s.d. increase in functional connectivity between the DMN or CEN and attention network or CEN, the risk of TN was reduced by 39.5% (IVW OR = 0.605, 95% CI: 0.403–0.909, *p* = 0.016). The functional connectivity between insula or cingulate and frontal regions was positively associated with the risk of TN, and 1 s.d. increase in the functional connectivity between the SN or DMN and SN or DMN increased TN risk by 53.9% (IVW OR = 1.539,95% CI: 1.018–2.324, *p* = 0.041). The functional connectivity between parietal and temporal regions was positively associated with the risk of TN, and 1 s.d. increase in the functional connectivity between the attention network or CEN or SN and DMN or CEN increased TN risk by 48.7% (IVW OR = 1.487, 95% CI: 1.070–2.067, *p* = 0.018).

### 3.4 Effects of Inflammatory Proteins on PHN and TN

**FIGURE 5:**
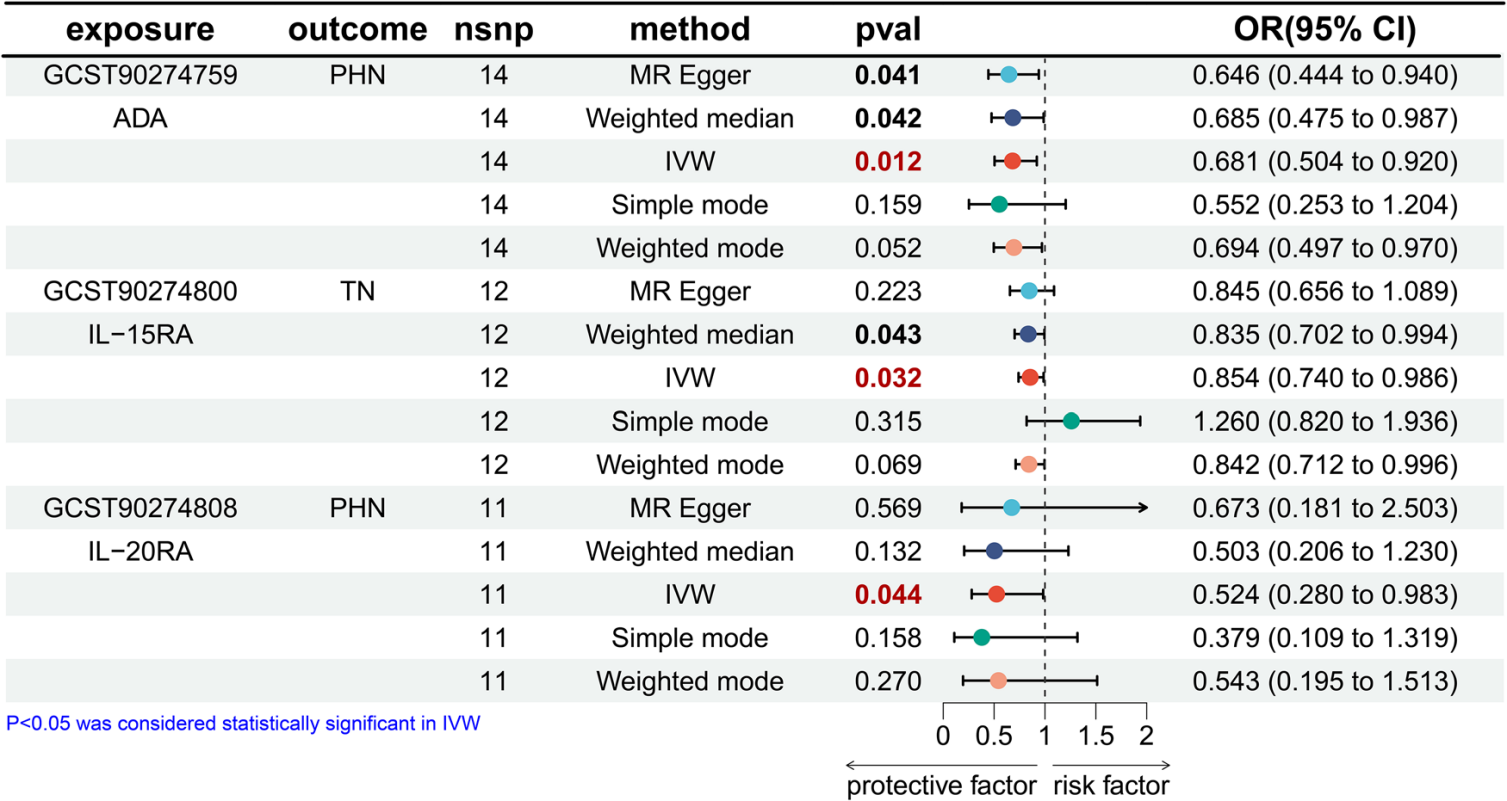
Mendelian randomization results of causal effects between inflammatory proteins to PHN and TN.

**FIGURE 6:**
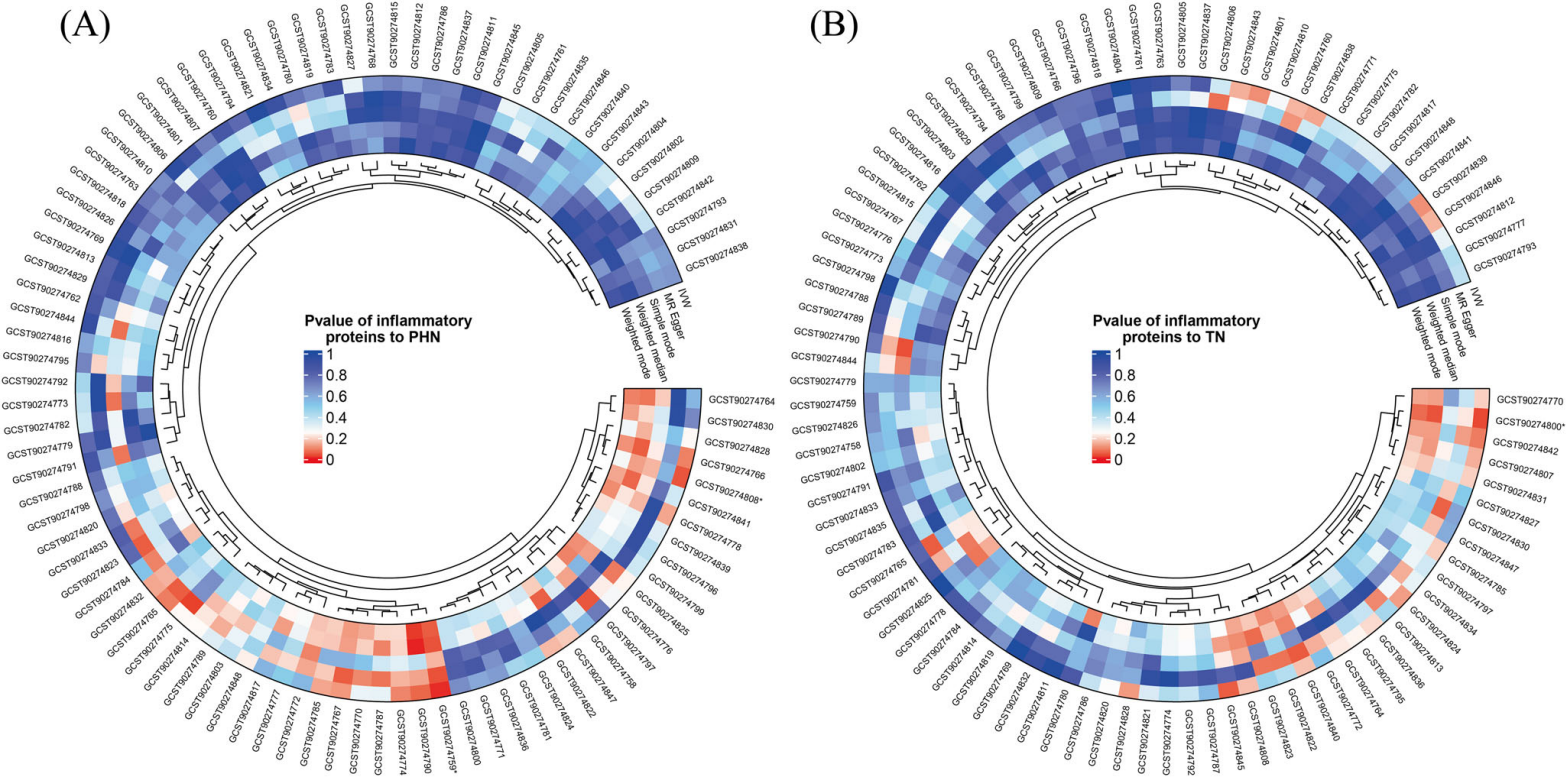
The heatmap internally displays a dendrogram mapping colours between 0‐1 based on the value of Pval, fading from red to blue, with the redder colour of the heatmap indicating a strongercausal relationship. The outer part of the circular heat map shows the GWAS number of the data, and positive results are marked with an *. The outermost circle of the heat map is the main research method IVW for our inclusion criteria, the five methods we labelled in the opening of the circle heat map.(A) *p*‐value of inflammatory proteins to PHN, (B) *p*‐value of inflammatory proteins to TN.

In the MR analysis of inflammatory proteins to PHN, we found that two inflammatory factors, adenosine deaminase (ADA) (IVW OR = 0.681, 95% CI: 0.504–0.920, *p* = 0.012) and IL‐20RA (IVW OR = 0.524 (95% CI: 0.280–0.983, *p* = 0.044) demonstrated an inverse correlation with PHN, while IL‐15RA (IVW OR = 0.854, 95% CI: 0.740–0.986, *p* = 0.032) exhibited an inverse correlation with TN (Figure 5), and we also visualized heatmaps for the forward analysis (Figure 6).

### 3.5 Sensitivity Analysis

Based on the MR‐PRESSO global test analysis and the MR‐Egger regression intercept method, it was demonstrated that there was no horizontal pleiotropy in the MR studies (*p* > 0.05, **Supplementary Material 2**: Tables S1–S3) and that genetic pleiotropy did not bias the results. Cochran's *Q* statistic with Rucker's *Q* statistic did not show heterogeneity (*p* > 0.05, Additional file 8: Table S7).

The results of the “leave‐one‐out” analysis proved the reliability of the MR analysis (the results were on the side of the zero line regardless of the exclusion of any of the SNPs (**Supplementary Material 1**: Figures S2, S5, S8, and S9). Scatter plot showing the overall effect of brain network function with inflammatory proteins on PHN and TN **(Supplementary Material 1**: Figures S3, S5, and S8). In addition, forest plots showed a causal relationship between brain network function and inflammatory proteins in PHN and TN (**Supplementary Material 1**: Figures S4, S7, and S9).

### 3.6 Mediation Analysis

**FIGURE 7:**
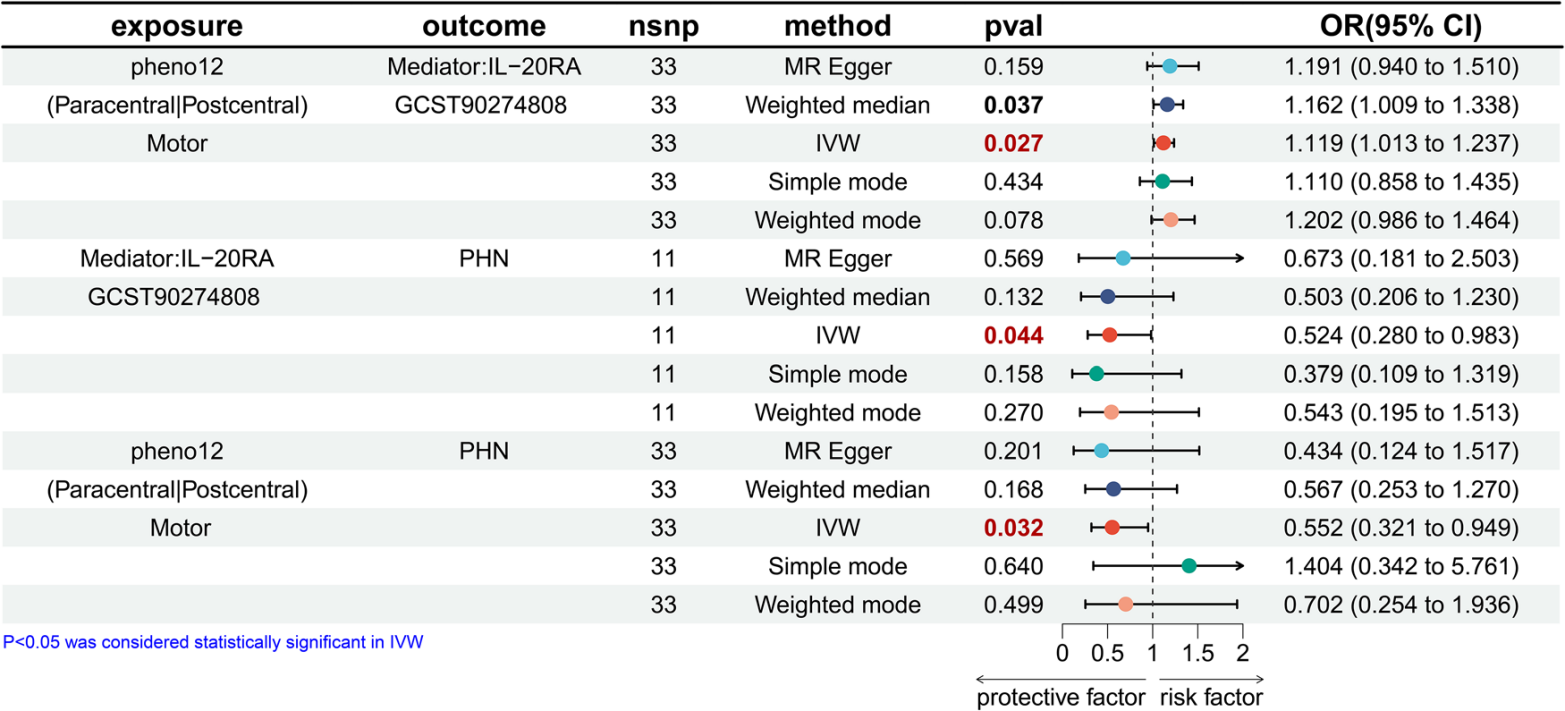
Mediation effect of brain rsfMRI phenotypes on PHN via IL‐20RA.

In this study, brain functional networks and circulating inflammatory proteins were both causally involved in PHN versus TN, and inflammatory factors may mediate the pathway between rsfMRI phenotypes and PHN versus TN. Consequently, three inflammatory factors with causal effects on PHN and TN, as well as brain network function, were included in the mediation analysis (Figure 7). In the motor network, the risk of developing IL‐20RA is increased by 11.9% for every 1 s.d. increase in amplitude traits (nodes) in the paracentral or postcentral region (IVW: odds ratio (OR) = 1.119, 95% CI: 1.013–1.237, *p* = 0.027). Mediation analysis showed that IL‐20RA displayed a significant mediating role between the pheno12 rsfMRI phenotype associated with Motor network and PHN *β* = ‐0.072, CI = [‐0.138–0.007], *p* = 0.029). The mediating proportion is 12.2%, and the direction of the effect is opposite to that of the independent variable.

## 4 Discussion

In this study, we used MR methods to explore 191 brain rsfMRI phenotypes and the causal effects of 91 inflammatory factors on two common pNPs. This study represents the first demonstration of a causal relationship between functional brain networks and pNP disease. The findings provide insights into the pathophysiology of the disease and suggest potential new non‐invasive therapeutic strategies. In therapeutic modalities such as transcranial magnetic stimulation and transcranial electrical stimulation, there exists the potential to precisely identify and target specific regional areas within the brain network for effective intervention. 11 related brain network phenotypes were identified as well as three inflammatory proteins associated with PHN and TN. In the motor network, the risk of developing PHN is reduced and the levels of IL‐20RA are increased for an increase in amplitude traits (nodes) in the paracentral or postcentral region. The inflammatory protein IL‐20RA partially mediates amplitude traits (nodes) in the paracentral or postcentral region in the motor network effect on PHN.

The concept of whole brain connectivity is now a prominent area of investigation in the field of pain research. Functional brain networks consist of a set of interconnected brain regions, not just individual brain regions, and advanced brain function relies on the collaboration of large‐scale networks rather than isolated individual regions. (Luchtmann et al. 2014; Reddan and Wager 2018) A number of studies have demonstrated that patients with PHN exhibit disturbances in the connectivity of the triple network (DMN, SN, CEN) as well as the affective, motor, and attentional networks. (Wu et al. 2020; Hong et al. 2018; Wu et al. 2022; De Ridder et al. 2022). The DMN is the neural basis of self‐awareness, with the medial prefrontal cortex (mPFC) and PCC serving as its primary anchoring structures. (Spreng and Grady 2010; Tu et al. 2019). Although the DMN does not directly process painful stimuli, it plays an important role in the long‐term perceptual and emotional experience of pain. In the case of persistent and chronic pain, pain‐related somatosensory cortical activity may become functionally connected to the DMN, thereby making pain an inherent part of self‐perception. (Alshelh et al. 2018). The DMN regions (LMTC, MFG, and PCC) are considered to be key nodes in the regulation of pain. The mPFC, which is a key component of the MFG, is thought to play a dual and opposite role in pain. (De Ridder et al. 2022; Wang et al. 2015) during mild pain, there was a relationship between catastrophizing and activity in cortical regions associated with affective, attentional, and motor aspects of pain, including dorsolateral prefrontal, insula, rostral anterior cingulate, premotor, and parietal cortices. (Seminowicz and Davis 2006) PHN also decreased gray matter volume in the right precentral gyrus. (Liu et al. 2019) The SN is responsible for identifying and integrating the significance of both internal and external stimuli, subsequently directing attention towards these stimuli deemed important. The role in pain is mainly in the allocation of attention to painful stimuli, emotional appraisal, and interaction with other brain networks, including the DMN and CEN. The capacity to rapidly identify painful stimuli as salient events and direct attention toward the source of the pain is primarily dependent on the anterior insula and dorsal anterior cingulate cortex. (Leknes et al. 2013; De Ridder et al. 2021) The disruption of the CEN, which is responsible for tasking and decision‐making and is known as the brain's “outer mind,” including the dorsolateral PFC and parietal cortex, may account for the combined cognitive dysfunction observed in pain patients. (Wade et al. 2011) The results of our MR analysis indicate that the networks of brain regions involved in motor‐related brain areas, such as the sensorimotor network (SMN) and the CEN, are closely related to the PHN. The sensorimotor network encompasses functional areas of the primary motor cortex, the cingulate cortex, the premotor cortex, and the paromotor areas. It also includes the primary and sensory cortex of the parietal lobe. In addition, we found that a decrease in paracentral or postcentral activity was associated with an increased risk of PHN, and reduced activity in the paracentral or frontal or supplementary motor area and frontal, and precuneus or angular or cingulate and frontal, cuneus or occipital and frontal regions was associated with a reduced risk of PHN. The risk of PHN is contingent upon an increase in the functional connectivity of the Attention Network, or SN, or the Motion Network‐CEN, or SN, DMN, or CEN and CEN, visual and CEN, which is accompanied by a corresponding increase in the risk of PHN. This MRI association is consistent with previous studies reporting abnormal brain network connectivity in patients with PHN. In summary, our MRI‐based evidence suggests that the triple network (DMN, SN, CEN) and motor, attentional, and visual networks may play an important role in PHN. Mediation studies have also found that the inflammatory factor IL‐20RA acts as a reverse mediator. IL‐20RA is a crucial component of the IL‐20 signaling pathway. It interacts with other receptor subunits in the IL‐20 signaling pathway (e.g., IL‐20RB) to jointly mediate IL‐20 signaling. IL‐20RA is highly expressed in the skin, which may be involved in epidermal function. It is closely related to the onset and progression of skin diseases and plays a key role in immune regulation and inflammatory processes. (Wang et al. 2006; Blumberg et al. 2001) In motor networks, an increase in amplitude features (nodes) in the paracentral or postcentral regions has been observed to result in elevated levels of IL‐20RA, which in turn serves to mediate a low risk of developing PHN. During the phase of nerve repair, IL‐20RA has been hypothesized to play a protective role through the “central motor network—peripheral immune—neuroprotective cross‐systems regulatory axis.” It is hypothesized that an increase in the efficiency of the motor network activates the hypothalamo‐pituitary‐adrenal axis, which in turn leads to an increase in cortisol release, an up‐regulation of IL‐20RA expression, and the promotion of regulatory T‐cell differentiation. These effects include the inhibition of neuroinflammation and the promotion of tissue repair, as well as pain relief. (Gao et al. 2021; Zhang et al. 2017).

The “Dynamic Pain Connectome” region (including the DMN / Cognitive Control Network / SN) plays a significant role in the pathophysiology of TN. (Zhang et al. 2021) The study revealed that the DMN, SMN, and SN exhibited distinct functional connectivity patterns in TN patients. Additionally, the severity of pain was negatively correlated with SN abnormalities. (Xu et al. 2022) In patients with TN, we found that the global functional connectivity of the triple network was also altered, with connections between SN and other networks (including SN or motor network and SN or DMN, SN or DMN and SN or DMN, and the Attention network or CEN or SN and DMN or CEN) all positively correlating with TN risk. Conversely, increased connectivity of motor and motor or attention networks was negatively associated with TN. In a previous study Wang et al. (2015) found that the local consistency of the left PCG was reduced in patients with TN and positively correlated with disease duration. Yan et el. (2019) also found reduced dynamic local coherence in the left PCG in TN patients by dynamic local coherence studies. The results of these studies have consistently demonstrated the presence of abnormal alterations in spontaneous neural activity in brain areas related to the motor system in patients with TN. Furthermore, gray matter volume was diminished in the primary somatosensory cortex, orbitofrontal cortex, secondary somatosensory cortex, thalamus, insula, anterior cingulate gyrus, cerebellum, and dorsolateral prefrontal cortex in patients with TN. Notably, the reduction in gray matter volume in the anterior cingulate gyrus and temporal lobe was associated with an increase in the duration of pain. (Obermann et al. 2013) Multiple functional regions of the insula‐related network were found to be involved in TN in our MR analysis, which is consistent with previous studies. In patients with TN, a significant decrease in localized gyration index (LGI) was observed in the left insular cortex, which was negatively correlated with pain intensity. Additionally, an increase in functional connectivity with the insula of the left posterior cingulate cortex and thalamus was noted, which was positively correlated with the course of the disease. These findings provide new evidence for the involvement of insular anomalies in the pathophysiology of TN (Wang et al. 2018; Wang et al. 2017). However, inflammatory factors were not found to play a mediating role.

This is the first study to perform a large‐scale MR analysis of the causal relationship between brain network function, inflammatory proteins, and several pNP subtypes. One of the key strengths of our study is the utilization of rsfMRI data for magnetic resonance analysis. In contrast to MRI, which is limited to reflecting the structure of a specific brain region, rsfMRI is capable of directly reflecting the degree of functional activity of a specific brain region and can reveal the intrinsic stabilization network of the human brain. Our study provides important clues to understand the aberrant functional connectivity of PHN with TN and takes mediation analysis to explore the role inflammatory proteins play in it. Advancing our understanding of pNP pathogenesis and developing predictive biomarkers for component processes associated with pain. The following limitations exist in this study. Firstly, as the dataset utilized is predominantly derived from European populations, the generalizability of our findings to other populations must be validated through the utilization of local GWAS data. Secondly, the 91 inflammatory proteins in question originate from the blood, not the cerebrospinal fluid. Finally, the small NP sample size must be meta‐analyzed with a larger data sample in order to provide further validation. Despite these limitations, to the best of our knowledge, this may be the first MR study to examine the causal relationship between rsfMRI phenotypes and pNP. This study demonstrates the causal relationship between functional brain networks and NP, as well as the mediating role played by inflammatory proteins.

## 5 Conclusion

In this study, we comprehensively explored the causal relationship between brain functional networks, circulating inflammatory proteins, and PHN and TN, and identified important brain functional networks that may be pathogenic for PHN and TN, as well as important brain regions for the development of new therapeutic agents. Two positive and three negative causal effects were identified between genetic susceptibility to rsfMRI phenotypes and PHN, and two negative causal effects between circulating inflammatory proteins and PHN. The inflammatory protein IL‐20RA functions as a mediator in the pathway from the phenotype Pheno 12 with the brain motor network to PHN. A total of six causal effects were identified between genetic susceptibility to fMRI and TN. Of these, three were positive and three were negative. Conversely, only one causal effect was identified between circulating inflammatory proteins and TN, which was negative. Furthermore, it appears that inflammatory proteins are not involved in the mediation of this process.

## Author Contributions

**Wen‐Hui Liu**: conceptualization, investigation, writing – original draft, methodology, formal analysis, software, resources, and visualization. **Hui‐Min Hu**: conceptualization, methodology, software anf data curation. **Chen Li**: conceptualization, methodology, and software. **Qing Shi**: conceptualization, methodology, and formal analysis. **Yi‐Fan Li**: conceptualization, data curation, supervision, resources, and project administration. **Peng Mao**: writing – review and editing and visualization. **Bi‐Fa Fan**: conceptualization, writing – review and editing, funding acquisition, and validation.

## Conflicts of Interest

The authors declare no conflicts of interest.

## Availability of Data and Materials

Our data were derived from publicly available GWASs, and 91 circulating inflammatory protein datasets were derived from the GWAS Catalog. https://www.ebi.ac.uk/gwas/studies; pNPs datasets were derived from the FinnGen biobank analysis round 11. https://r11.finngen.fi/.

## Peer Review

The peer review history for this article is available at https://publons.com/publon/10.1002/brb3.70751.

## Clinical Trial Number

The authors have nothing to report.

## Ethics Statement

The authors have nothing to report.

## Consent

The authors have nothing to report on consent to participation and publication.

## Supporting information




**Supplementary Figures**: brb370751‐sup‐0001‐FigureS1‐S9.docx

**Supplementary Tables**: brb370751‐sup‐0001‐TableS1‐S3.xlsx
Supporting Information

## Data Availability

Data sharing is not applicable to this article as no new data were created or analyzed in this study.

## References

[brb370751-bib-0001] Alhajri, N. , S. A. Boudreau , and T. Graven‐Nielsen . 2023. “Decreased Default Mode Network Connectivity Following 24 H of Capsaicin‐induced Pain Persists During Immediate Pain Relief and Facilitation.” The Journal of Pain 24, no. 5: 796–811. 10.1016/j.jpain.2022.12.004. 36521671

[brb370751-bib-0002] Alshelh, Z. , K. K. Marciszewski , R. Akhter , et al. 2018. “Disruption of Default Mode Network Dynamics in Acute and Chronic Pain States.” NeuroImage: Clinical 17: 222–231. 10.1016/j.nicl.2017.10.019. 29159039 PMC5683191

[brb370751-bib-0003] Araya, E. I. , R. F. Claudino , E. J. Piovesan , and J. G. Chichorro . 2020. “Trigeminal Neuralgia: Basic and Clinical Aspects.” Current Neuropharmacology 18, no. 2: 109–119. 10.2174/1570159;17666191010094350. 31608834 PMC7324879

[brb370751-bib-0004] Baliki, M. N. , P. Y. Geha , A. V. Apkarian , and D. R. Chialvo . 2008. “Beyond Feeling: Chronic Pain Hurts the Brain, Disrupting the Default‐mode Network Dynamics.” Journal of Neuroscience 28, no. 6: 1398–1403. 10.1523/jneurosci.4123-07.2008. 18256259 PMC6671589

[brb370751-bib-0005] Baron, R. , A. Binder , and G. Wasner . 2010. “Neuropathic Pain: Diagnosis, Pathophysiological Mechanisms, and Treatment.” Lancet Neurology 9, no. 8: 807–819. 10.1016/s1474-4422(10)70143-5. 20650402

[brb370751-bib-0006] Bendtsen, L. , J. M. Zakrzewska , T. B. Heinskou , et al. 2020. “Advances in Diagnosis, Classification, Pathophysiology, and Management of Trigeminal Neuralgia.” Lancet Neurology 19, no. 9: 784–796. 10.1016/s1474-4422(20)30233-7. 32822636

[brb370751-bib-0007] Blumberg, H. , D. Conklin , W. F. Xu , et al. 2001. “Interleukin 20: Discovery, Receptor Identification, and Role in Epidermal Function.” Cell 104, no. 1: 9–19. 10.1016/s0092-8674(01)00187-8. 11163236

[brb370751-bib-0008] Bowden, J. , and M. V. Holmes . 2019. “Meta‐Analysis and Mendelian Randomization: A Review.” Research Synthesis Methods 10, no. 4: 486–496. 10.1002/jrsm.1346. 30861319 PMC6973275

[brb370751-bib-0009] Burgess, S. , D. S. Small , and S. G. Thompson . 2017. “A Review of Instrumental Variable Estimators for Mendelian Randomization.” Statistical Methods in Medical Research 26, no. 5: 2333–2355. 10.1177/0962280215597579. 26282889 PMC5642006

[brb370751-bib-0010] Burgess, S. , and S. G. Thompson . 2017. “Interpreting Findings From Mendelian Randomization Using the MR‐Egger Method.” European Journal of Epidemiology 32, no. 5: 377–389. 10.1007/s10654-017-0255-x. 28527048 PMC5506233

[brb370751-bib-0011] Cauda, F. , F. D'Agata , and K. Sacco , et al. 2010. “Altered Resting State Attentional Networks in Diabetic Neuropathic Pain.” Journal of Neurology, Neurosurgery, and Psychiatry 81, no. 7: 806–811. 10.1136/jnnp.2009.188631. 19955113

[brb370751-bib-0012] Cohen, J. F. , M. Chalumeau , R. Cohen , D. A. Korevaar , B. Khoshnood , and P. M. Bossuyt . 2015. “Cochran's *Q* Test Was Useful to Assess Heterogeneity in Likelihood Ratios in Studies of Diagnostic Accuracy.” Journal of Clinical Epidemiology 68, no. 3: 299–306. 10.1016/j.jclinepi.2014.09.005. 25441698

[brb370751-bib-0013] Cohen, S. P. , and J. Mao . 2014. “Neuropathic Pain: Mechanisms and Their Clinical Implications.” BMJ 348: f7656. 10.1136/bmj.f7656. 24500412

[brb370751-bib-0014] Davies, N. M. , M. V. Holmes , and G. Davey Smith . 2018. “Reading Mendelian Randomisation Studies: A Guide, Glossary, and Checklist for Clinicians.” BMJ 362: k601. 10.1136/bmj.k601. 30002074 PMC6041728

[brb370751-bib-0015] de Ridder, D. , D. Adhia , and S. Vanneste . 2021. “The Anatomy of Pain and Suffering in the Brain and Its Clinical Implications.” Neuroscience and Biobehavioral Reviews 130: 125–146. 10.1016/j.neubiorev.2021.08.013. 34411559

[brb370751-bib-0016] de Ridder, D. , S. Vanneste , M. Smith , and D. Adhia . 2022. “Pain and the Triple Network Model.” Frontiers in Neurology 13: 757241. 10.3389/fneur.2022.757241. 35321511 PMC8934778

[brb370751-bib-0017] Dworkin, R. H. 2002. “An Overview of Neuropathic Pain: Syndromes, Symptoms, Signs, and Several Mechanisms.” Clinical Journal of Pain 18, no. 6: 343–349. 10.1097/00002508-200211000-00001. 12441827

[brb370751-bib-0018] Elliott, L. T. , K. Sharp , F. Alfaro‐Almagro , et al. 2018. “Genome‐wide Association Studies of Brain Imaging Phenotypes in UK Biobank.” Nature 562, no. 7726: 210–216. 10.1038/s41586-018-0571-7. 30305740 PMC6786974

[brb370751-bib-0019] Emdin, C. A. , A. V. Khera , and S. Kathiresan . 2017. “Mendelian Randomization.” JAMA 318, no. 19: 1925–1926. 10.1001/jama.2017.17219. 29164242

[brb370751-bib-0020] Finn, E. S. , X. Shen , D. Scheinost , et al. 2015. “Functional Connectome Fingerprinting: Identifying Individuals Using Patterns of Brain Connectivity.” Nature Neuroscience 18, no. 11: 1664–1671. 10.1038/nn.4135. 26457551 PMC5008686

[brb370751-bib-0021] Gao, W. , H. Wen , L. Liang , et al. 2021. “IL20RA signaling Enhances Stemness and Promotes the Formation of an Immunosuppressive Microenvironment in Breast Cancer.” Theranostics 11, no. 6: 2564–2580. 10.7150/thno.45280. 33456560 PMC7806486

[brb370751-bib-0022] Gopinath, K. , W. Ringe , A. Goyal , et al. 2011. “Striatal Functional Connectivity Networks are Modulated by fMRI Resting State Conditions.” NeuroImage 54, no. 1: 380–388. 10.1016/j.neuroimage.2010.07.021. 20637878

[brb370751-bib-0023] Grasby, K. L. , N. Jahanshad , J. N. Painter , et al. 2020. “The Genetic Architecture of the Human Cerebral Cortex.” Science 367, no. 6484: eaay6690. 10.1126/science.aay6690. 32193296 PMC7295264

[brb370751-bib-0024] Hong, S. , L. Gu , F. Zhou , et al. 2018. “Altered Functional Connectivity Density in Patients With Herpes Zoster and Postherpetic Neuralgia.” Journal of Pain and Research 11: 881–888. 10.2147/jpr.S154314. PMC5931198 29740216

[brb370751-bib-0025] Jensen, T. S. , R. Baron , M. Haanpää , et al. 2011. “A New Definition of Neuropathic Pain.” Pain 152, no. 10: 2204–2205. 10.1016/j.pain.2011.06.017. 21764514

[brb370751-bib-0026] Ji, R. R. , A. Chamessian , and Y. Q. Zhang . 2016. “Pain Regulation by Non‐neuronal Cells and Inflammation.” Science 354, no. 6312: 572–577. 10.1126/science.aaf8924. 27811267 PMC5488328

[brb370751-bib-0027] Kamat, M. A. , J. A. Blackshaw , R. Young , et al. 2019. “PhenoScanner V2: An Expanded Tool for Searching Human Genotype‐phenotype Associations.” Bioinformatics 35, no. 22: 4851–4853. 10.1093/bioinformatics/btz469. 31233103 PMC6853652

[brb370751-bib-0028] Kim, C. , J. Kim , H. Chang , D. Hong , S. Hong , and H. Moon . 2023. “Longitudinal Change in Brain Functional Connectivity With Herpes Zoster Patients: Neuroimaging Case Series.” Medicina 59, no. 6: 1045. 10.3390/medicina59061045. 37374249 PMC10303063

[brb370751-bib-0029] Leknes, S. , C. Berna , M. C. Lee , G. D. Snyder , G. Biele , and I. Tracey . 2013. “The Importance of Context: When Relative Relief Renders Pain Pleasant.” Pain 154, no. 3: 402–410. 10.1016/j.pain.2012.11.018. 23352758 PMC3590449

[brb370751-bib-0030] Liu, J. , L. Gu , Q. Huang , et al. 2019. “Altered Gray Matter Volume in Patients With Herpes Zoster and Postherpetic Neuralgia.” Journal of Pain and Research 12: 605–616. 10.2147/jpr.S183561. PMC6369852 30799946

[brb370751-bib-0031] Liu, J. , Y. Hao , M. Du , et al. 2013. “Quantitative Cerebral Blood Flow Mapping and Functional Connectivity of Postherpetic Neuralgia Pain: A Perfusion fMRI Study.” Pain 154, no. 1: 110–118. 10.1016/j.pain.2012.09.016. 23140909

[brb370751-bib-0032] Loeser, J. D. , and R. D. Treede . 2008. “The Kyoto Protocol of IASP Basic Pain Terminology.” Pain 137, no. 3: 473–477. 10.1016/j.pain.2008.04.025. 18583048

[brb370751-bib-0033] Luchtmann, M. , Y. Steinecke , S. Baecke , et al. 2014. “Structural Brain Alterations in Patients With Lumbar Disc Herniation: A Preliminary Study.” PLoS ONE 9, no. 3: e90816. 10.1371/journal.pone.0090816. 24595036 PMC3940958

[brb370751-bib-0034] Maarbjerg, S. , A. Gozalov , J. Olesen , and L. Bendtsen . 2014. “Trigeminal Neuralgia–A Prospective Systematic Study of Clinical Characteristics in 158 Patients.” Headache 54, no. 10: 1574–1582. 10.1111/head.12441. 25231219

[brb370751-bib-0035] Nemirovsky, I. E. , N. J. M. Popiel , J. Rudas , et al. 2023. “An Implementation of Integrated Information Theory in Resting‐State fMRI.” Communications Biology 6, no. 1: 692. 10.1038/s42003-023-05063-y. 37407655 PMC10322831

[brb370751-bib-0036] Nieto‐Rostro, M. , K. Ramgoolam , W. S. Pratt , A. Kulik , and A. C. Dolphin . 2018. “Ablation of α_2_δ‐1 Inhibits Cell‐surface Trafficking of Endogenous N‐type Calcium Channels in the Pain Pathway in Vivo.” Proceedings of the National Academy of Sciences of the United States of America 115, no. 51: E12043–E12052. 10.1073/pnas.1811212115. 30487217 PMC6305000

[brb370751-bib-0037] Oaklander, A. L. 2001. “The Density of Remaining Nerve Endings in Human Skin With and Without Postherpetic Neuralgia After Shingles.” Pain 92, no. 1–2: 139–145. 10.1016/s0304-3959(00)00481-4. 11323135

[brb370751-bib-0038] Obermann, M. , R. Rodriguez‐Raecke , S. Naegel , et al. 2013. “Gray Matter Volume Reduction Reflects Chronic Pain in Trigeminal Neuralgia.” NeuroImage 74: 352–358. 10.1016/j.neuroimage.2013.02.029. 23485849

[brb370751-bib-0039] Qiu, J. , M. Du , J. Yang , et al. 2021. “The Brain's Structural Differences Between Postherpetic Neuralgia and Lower Back Pain.” Scientific Reports 11, no. 1: 22455. 10.1038/s41598-021-01915-x. 34789811 PMC8599674

[brb370751-bib-0040] Reddan, M. C. , and T. D. Wager . 2018. “Modeling Pain Using fMRI: From Regions to Biomarkers.” Neuroscience Bulletin 34, no. 1: 208–215. 10.1007/s12264-017-0150-1. 28646349 PMC5799128

[brb370751-bib-0041] Scholz, J. , N. B. Finnerup , N. Attal , et al. 2019. “The IASP Classification of Chronic Pain for ICD‐11: Chronic Neuropathic Pain.” Pain 160, no. 1: 53–59. 10.1097/j.pain.0000000000001365. 30586071 PMC6310153

[brb370751-bib-0042] Seifert, F. , and C. Maihöfner . 2009. “Central Mechanisms of Experimental and Chronic Neuropathic Pain: Findings From Functional Imaging Studies.” Cellular and Molecular Life Sciences 66, no. 3: 375–390. 10.1007/s00018-008-8428-0. 18791842 PMC11131450

[brb370751-bib-0043] Seminowicz, D. A. , and K. D. Davis . 2006. “Cortical Responses to Pain in Healthy Individuals Depends on Pain Catastrophizing.” Pain 120, no. 3: 297–306. 10.1016/j.pain.2005.11.008. 16427738

[brb370751-bib-0044] Sommer, C. , M. Leinders , and N. Üçeyler . 2018. “Inflammation in the Pathophysiology of Neuropathic Pain.” Pain 159, no. 3: 595–602. 10.1097/j.pain.0000000000001122. 29447138

[brb370751-bib-0045] Spreng, R. N. , and C. L. Grady . 2010. “Patterns of Brain Activity Supporting Autobiographical Memory, Prospection, and Theory of Mind, and Their Relationship to the Default Mode Network.” Journal of Cognitive Neuroscience 22, no. 6: 1112–1123. 10.1162/jocn.2009.21282. 19580387

[brb370751-bib-0046] Tu, Y. , M. Jung , R. L. Gollub , et al. 2019. “Abnormal Medial Prefrontal Cortex Functional Connectivity and Its Association With Clinical Symptoms in Chronic Low Back Pain.” Pain 160, no. 6: 1308–1318. 10.1097/j.pain.0000000000001507. 31107712 PMC6530583

[brb370751-bib-0047] van den Heuvel, M. P. , and H. E. Hulshoff Pol . 2010. “Exploring the Brain Network: A Review on Resting‐State fMRI Functional Connectivity.” European Neuropsychopharmacology 20, no. 8: 519–534. 10.1016/j.euroneuro.2010.03.008. 20471808

[brb370751-bib-0048] Verbanck, M. , C. Y. Chen , B. Neale , and R. Do . 2018. “Detection of Widespread Horizontal Pleiotropy in Causal Relationships Inferred From Mendelian Randomization Between Complex Traits and Diseases.” Nature Genetics 50, no. 5: 693–698. 10.1038/s41588-018-0099-7. 29686387 PMC6083837

[brb370751-bib-0049] Wade, J. B. , D. L. Riddle , D. D. Price , and L. Dumenci . 2011. “Role of Pain Catastrophizing During Pain Processing in a Cohort of Patients With Chronic and Severe Arthritic Knee Pain.” Pain 152, no. 2: 314–319. 10.1016/j.pain.2010.10.034. 21130571

[brb370751-bib-0050] Wang, F. , E. Lee , M. A. Lowes , et al. 2006. “Prominent Production of IL‐20 by CD68+/CD11c+ Myeloid‐derived Cells in Psoriasis: Gene Regulation and Cellular Effects.” Journal of Investigative Dermatology 126, no. 7: 1590–1599. 10.1038/sj.jid.5700310. 16645593

[brb370751-bib-0051] Wang, Y. , D. Y. Cao , B. Remeniuk , S. Krimmel , D. A. Seminowicz , and M. Zhang . 2017. “Altered Brain Structure and Function Associated With Sensory and Affective Components of Classic Trigeminal Neuralgia.” Pain 158, no. 8: 1561–1570. 10.1097/j.pain.0000000000000951. 28520647

[brb370751-bib-0052] Wang, Y. , X. Zhang , Q. Guan , L. Wan , Y. Yi , and C. F. Liu . 2015. “Altered Regional Homogeneity of Spontaneous Brain Activity in Idiopathic Trigeminal Neuralgia.” Neuropsychiatric Disease and Treatment 11: 2659–2666. 10.2147/ndt.S94877. 26508861 PMC4610767

[brb370751-bib-0053] Wang, Y. , Y. Zhang , J. Zhang , et al. 2018. “Structural and Functional Abnormalities of the Insular Cortex in Trigeminal Neuralgia: A Multimodal Magnetic Resonance Imaging Analysis.” Pain 159, no. 3: 507–514. 10.1097/j.pain.0000000000001120. 29200179

[brb370751-bib-0054] Wang, Z. , Z. Zhao , Z. Song , et al. 2024. “Functional Alterations of the Brain Default Mode Network and Somatosensory System in Trigeminal Neuralgia.” Scientific Reports 14, no. 1: 10205. 10.1038/s41598-024-60273-6. 38702383 PMC11068897

[brb370751-bib-0055] Weizman, L. , L. Dayan , S. Brill , et al. 2018. “Cannabis Analgesia in Chronic Neuropathic Pain Is Associated With Altered Brain Connectivity.” Neurology 91, no. 14: e1285–e1294. 10.1212/wnl.0000000000006293. 30185448 PMC6177269

[brb370751-bib-0056] Williams, J. A. , S. Burgess , J. Suckling , et al. 2022. “Inflammation and Brain Structure in Schizophrenia and Other Neuropsychiatric Disorders: A Mendelian Randomization Study.” JAMA Psychiatry 79, no. 5: 498–507. 10.1001/jamapsychiatry.2022.0407. 35353173 PMC8968718

[brb370751-bib-0057] Wu, Y. , C. Wang , W. Qian , et al. 2020. “Disrupted Default Mode Network Dynamics in Recuperative Patients of Herpes Zoster Pain.” CNS Neuroscience & Therapeutics 26, no. 12: 1278–1287. 10.1111/cns.13433. 32677342 PMC7702236

[brb370751-bib-0058] Wu, Y. , C. Wang , L. Yu , et al. 2022. “Abnormal Within‐ and Cross‐Networks Functional Connectivity in Different Outcomes of Herpes Zoster Patients.” Brain Imaging and Behavior 16, no. 1: 366–378. 10.1007/s11682-021-00510-y. 34549378

[brb370751-bib-0059] Xu, H. , D. A. Seminowicz , S. R. Krimmel , M. Zhang , L. Gao , and Y. Wang . 2022. “Altered Structural and Functional Connectivity of Salience Network in Patients With Classic Trigeminal Neuralgia.” The Journal of Pain 23, no. 8: 1389–1399. 10.1016/j.jpain.2022.02.012. 35381362

[brb370751-bib-0060] Yan, J. , M. Li , S. Fu , et al. 2019. “Alterations of Dynamic Regional Homogeneity in Trigeminal Neuralgia: A Resting‐State fMRI Study.” Frontiers in Neurology 10: 1083. 10.3389/fneur.2019.01083. 31649618 PMC6794683

[brb370751-bib-0061] Yao, S. , M. Zhang , S. S. Dong , et al. 2022. “Bidirectional Two‐sample Mendelian Randomization Analysis Identifies Causal Associations Between Relative Carbohydrate Intake and Depression.” Nature Human Behaviour 6, no. 11: 1569–1576. 10.1038/s41562-022-01412-9. 35851841

[brb370751-bib-0062] Zhang, P. , Y. Jiang , G. Liu , et al. 2021. “Altered Brain Functional Network Dynamics in Classic Trigeminal Neuralgia: A Resting‐State Functional Magnetic Resonance Imaging Study.” Journal of Headache and Pain 22, no. 1: 147. 10.1186/s10194-021-01354-z. 34895135 PMC8903588

[brb370751-bib-0063] Zhang, W. , S. Magadi , Z. Li , et al. 2017. “IL‐20 Promotes Epithelial Healing of the Injured Mouse Cornea.” Experimental Eye Research 154: 22–29. 10.1016/j.exer.2016.11.006. 27818315 PMC5359042

[brb370751-bib-0064] Zhao, B. , T. Li , S. M. Smith , et al. 2022. “Common Variants Contribute to Intrinsic Human Brain Functional Networks.” Nature Genetics 54, no. 4: 508–517. 10.1038/s41588-022-01039-6. 35393594 PMC11987081

[brb370751-bib-0065] Zhao, B. , T. Luo , T. Li , et al. 2019. “Genome‐wide Association Analysis of 19,629 Individuals Identifies Variants Influencing Regional Brain Volumes and Refines Their Genetic co‐architecture With Cognitive and Mental Health Traits.” Nature Genetics 51, no. 11: 1637–1644. 10.1038/s41588-019-0516-6. 31676860 PMC6858580

[brb370751-bib-0066] Zhao, J. H. , D. Stacey , N. Eriksson , et al. 2023. “Genetics of Circulating Inflammatory Proteins Identifies Drivers of Immune‐mediated Disease Risk and Therapeutic Targets.” Nature Immunology 24, no. 9: 1540–1551. 10.1038/s41590-023-01588-w. 37563310 PMC10457199

[brb370751-bib-0067] Zuber, V. , N. F. Grinberg , D. Gill , et al. 2022. “Combining Evidence From Mendelian Randomization and Colocalization: Review and Comparison of Approaches.” American Journal of Human Genetics 109, no. 5: 767–782. 10.1016/j.ajhg.2022.04.001. 35452592 PMC7612737

