# Supplementary material for: Mendelian Randomization Analysis Reveal the Role of Circulating Inflammatory Proteins in Mediating Functional Brain Networks and Peripheral Neuropathic Pain Effects
Source: Brain Behav. 2025 Aug 22;15(8):e70751. doi: 10.1002/brb3.70751 (PMC12373714; doi:10.1002/brb3.70751)
Supplement: Supplementary file 1 — Supplementary Figures: brb370751‐sup‐0001‐FigureS1‐S9.docx [file BRB3-15-e70751-s002.docx]

**Additional Figures**

**Figure S1.** Forest plots for the effect of PHN and TN on brain functional networks.

**Figure S2.** MR leave-one-out sensitivity analysis for brain functional networks on PHN.

**Figure S3.** Scatter plots for the effect of brain functional networks on PHN.

**Figure S4.** Forest plots for the effect of brain functional networks on PHN.

**Figure S5.** MR leave-one-out sensitivity analysis for brain functional networks on TN.

**Figure S6.** Scatter plots for the effect of brain functional networks on TN.

**Figure S7.** Forest plots for the effect of brain functional networks on TN.

**Figure S8.** Scatter plots,MR leave-one-out sensitivity analysis and Forest plots for the effect of inflammatory proteins on PHN and TN.

**Figure S9.** Scatter plots,MR leave-one-out sensitivity analysis and Forest plots for the effect of brain functional network ( Phneo12 ) on inflammatory protein ( IL20RA ).

**Figure S1.** Forest plots for the effect of PHN and TN on brain functional networks.

**
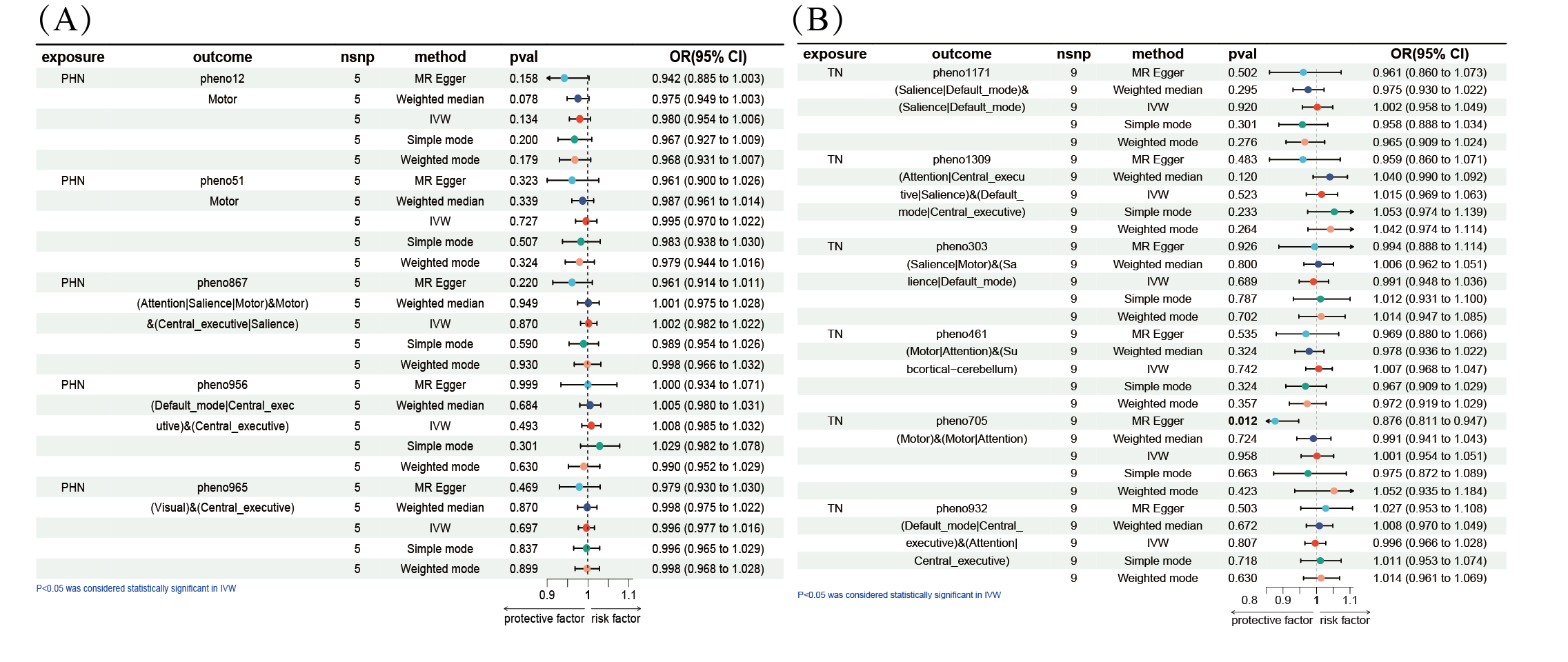
**

1. MR effect size for PHN on brain functional networks.
2. MR effect size for TN on brain functional networks.

**Figure S2.** MR leave-one-out sensitivity analysis for brain functional networks on PHN.


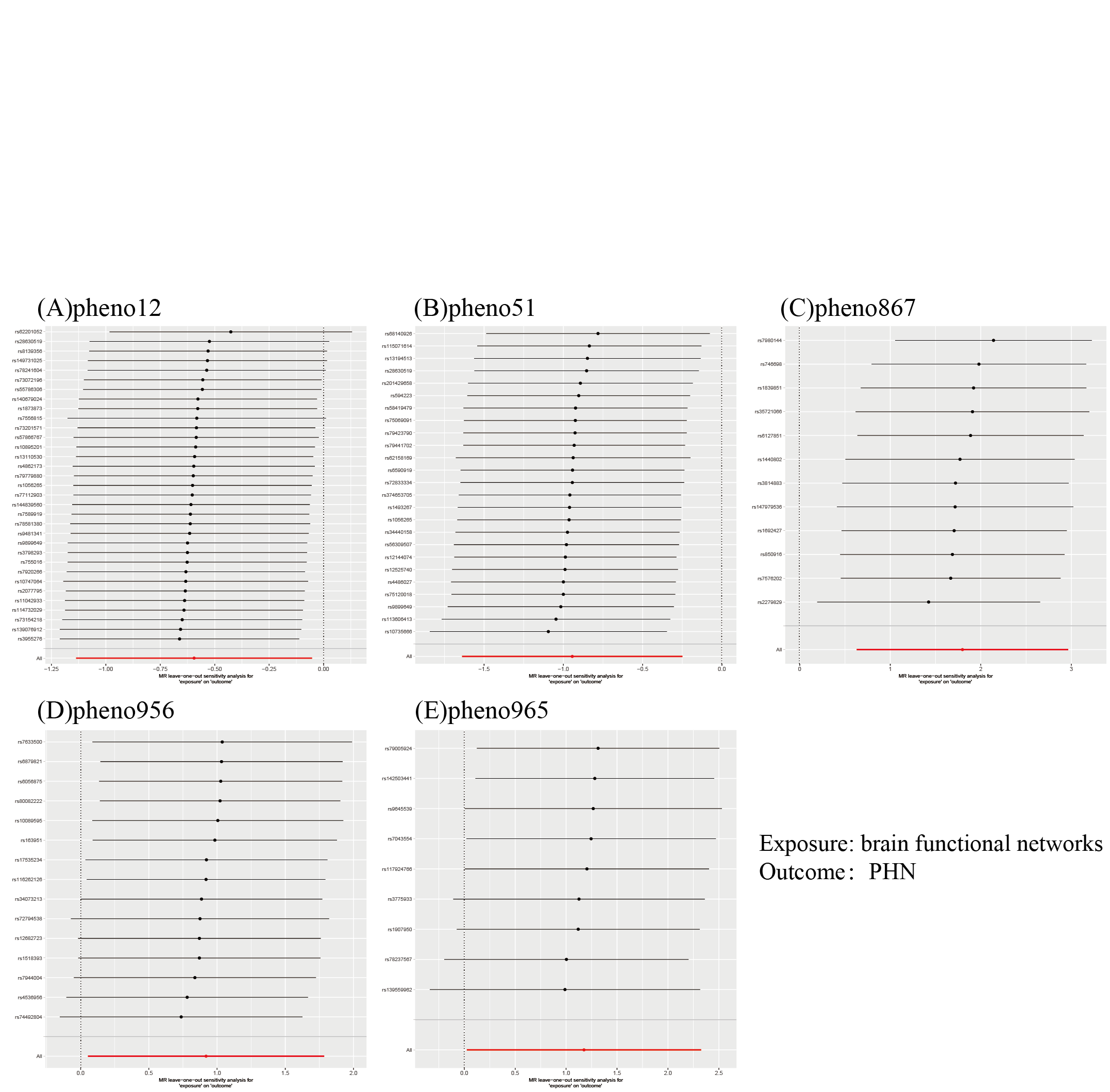


1. Analysis for "pheno12" on "PHN"
2. Analysis for "pheno51" on "PHN"
3. Analysis for "pheno867" on "PHN"
4. Analysis for "pheno956" on "PHN"
5. Analysis for "pheno965" on "PHN"

**Figure S3.**Scatter plots for the effect of brain functional networks on PHN.


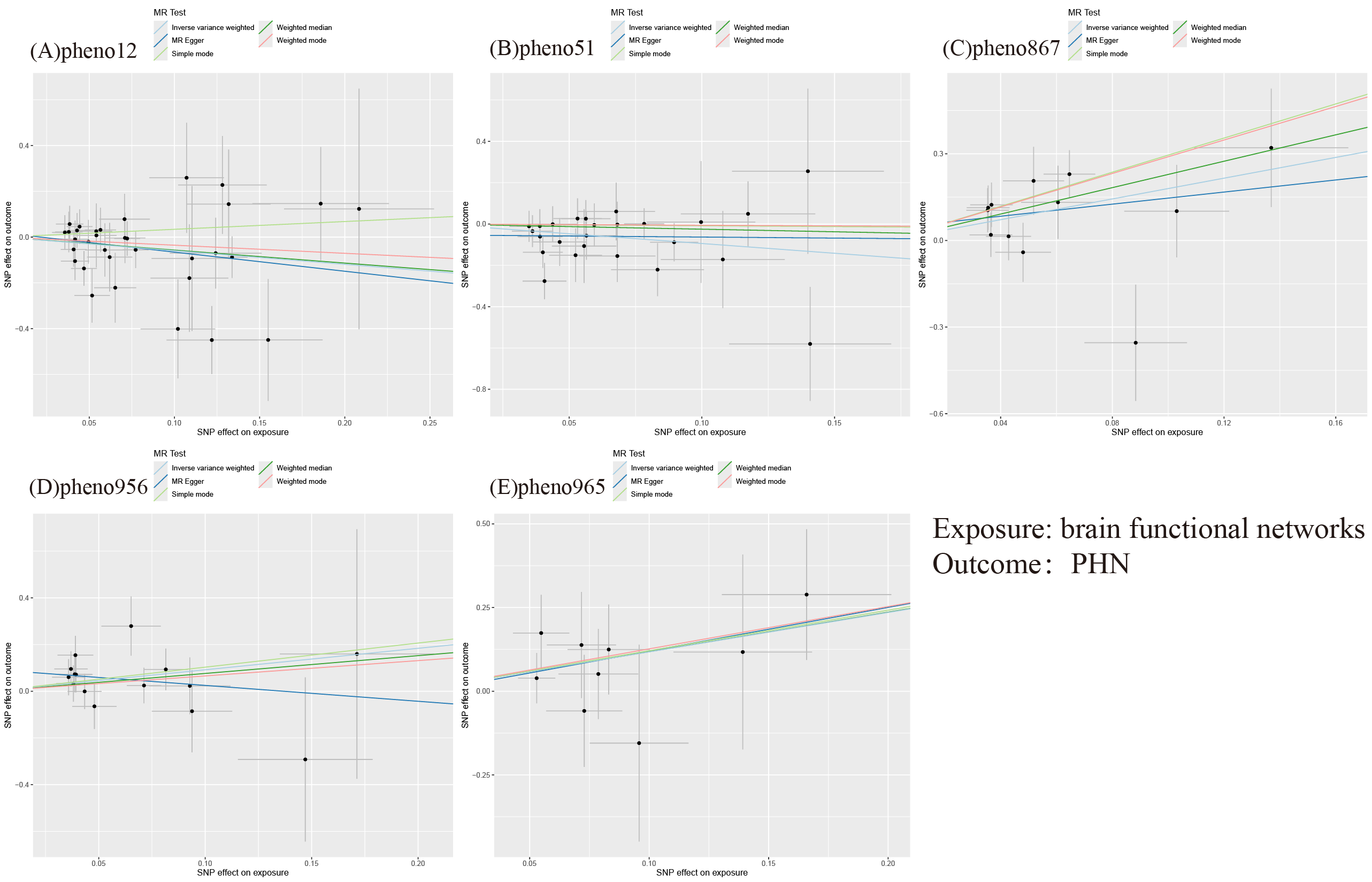


1. Scatter plots for "pheno12" on "PHN"
2. Scatter plots for "pheno51" on "PHN"
3. Scatter plots for "pheno867" on "PHN"
4. Scatter plots for "pheno956" on "PHN"
5. Scatter plots for "pheno965" on "PHN"

**Figure S4.**Forest plots for the effect of brain functional networks on PHN.


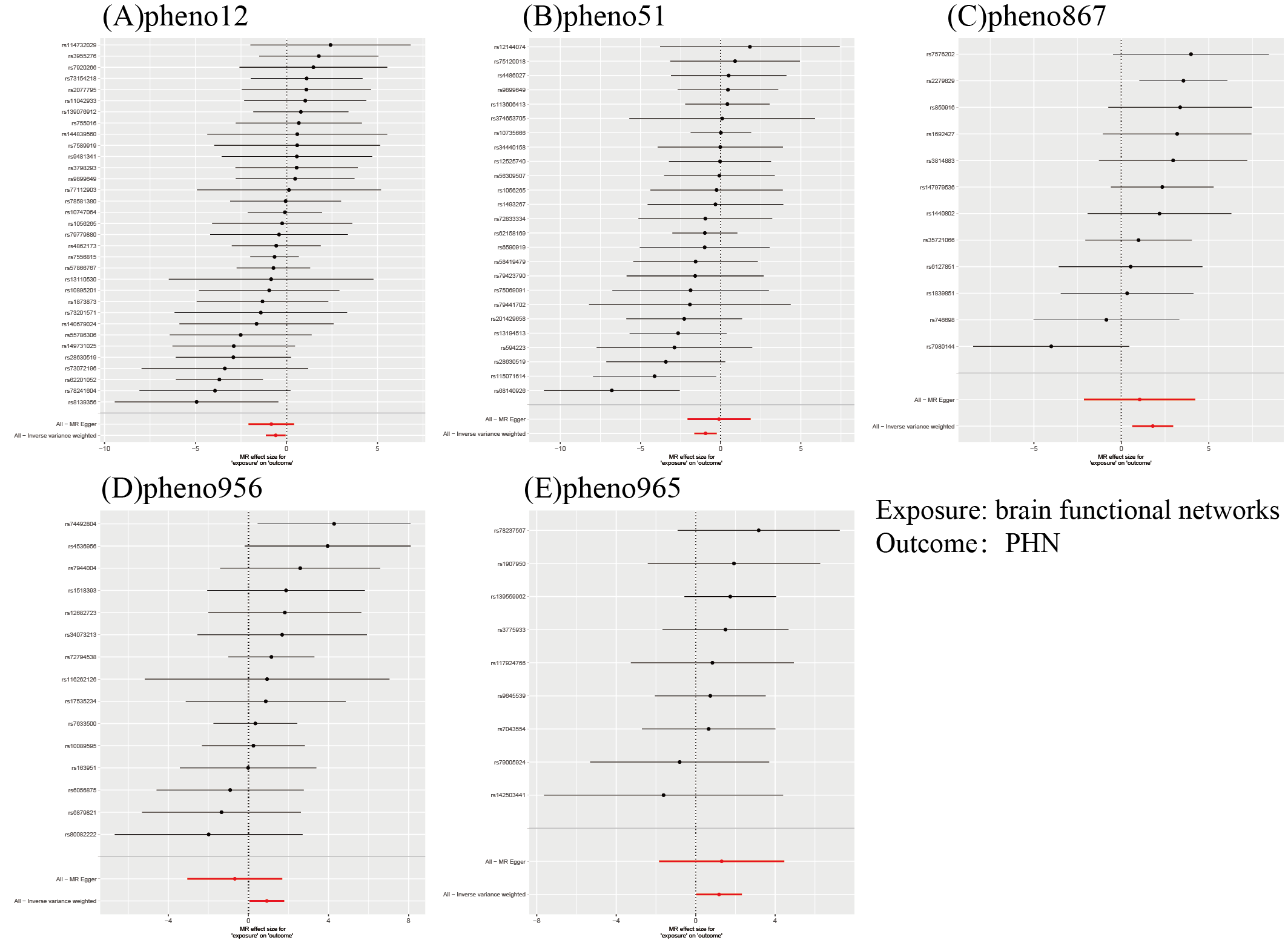


1. MR effect size for "pheno12" on "PHN"
2. MR effect size for "pheno51" on "PHN"
3. MR effect size for "pheno867" on "PHN"
4. MR effect size for "pheno956" on "PHN"
5. MR effect size for "pheno965" on "PHN

**Figure S5.** MR leave-one-out sensitivity analysis for brain functional networks on TN.


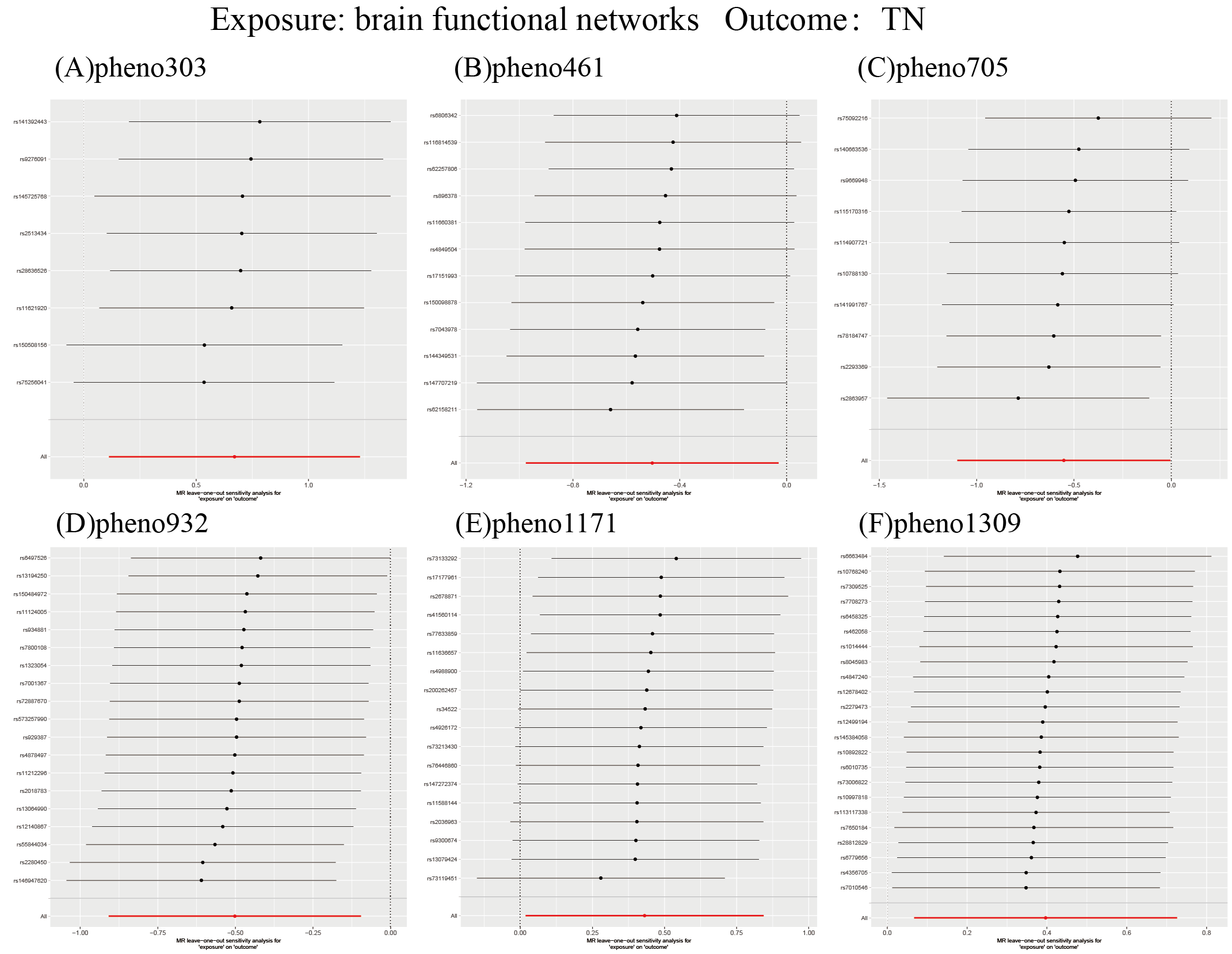


1. Analysis "pheno303" on "TN"
2. Analysis for "pheno461" on "TN"
3. Analysis for "pheno705" on "TN"
4. Analysis for "pheno932" on "TN"
5. Analysis for "pheno1171" on "TN"
6. Analysis for "pheno1309" on "TN

**Figure S6.** Scatter plots for the effect of brain functional networks on TN.


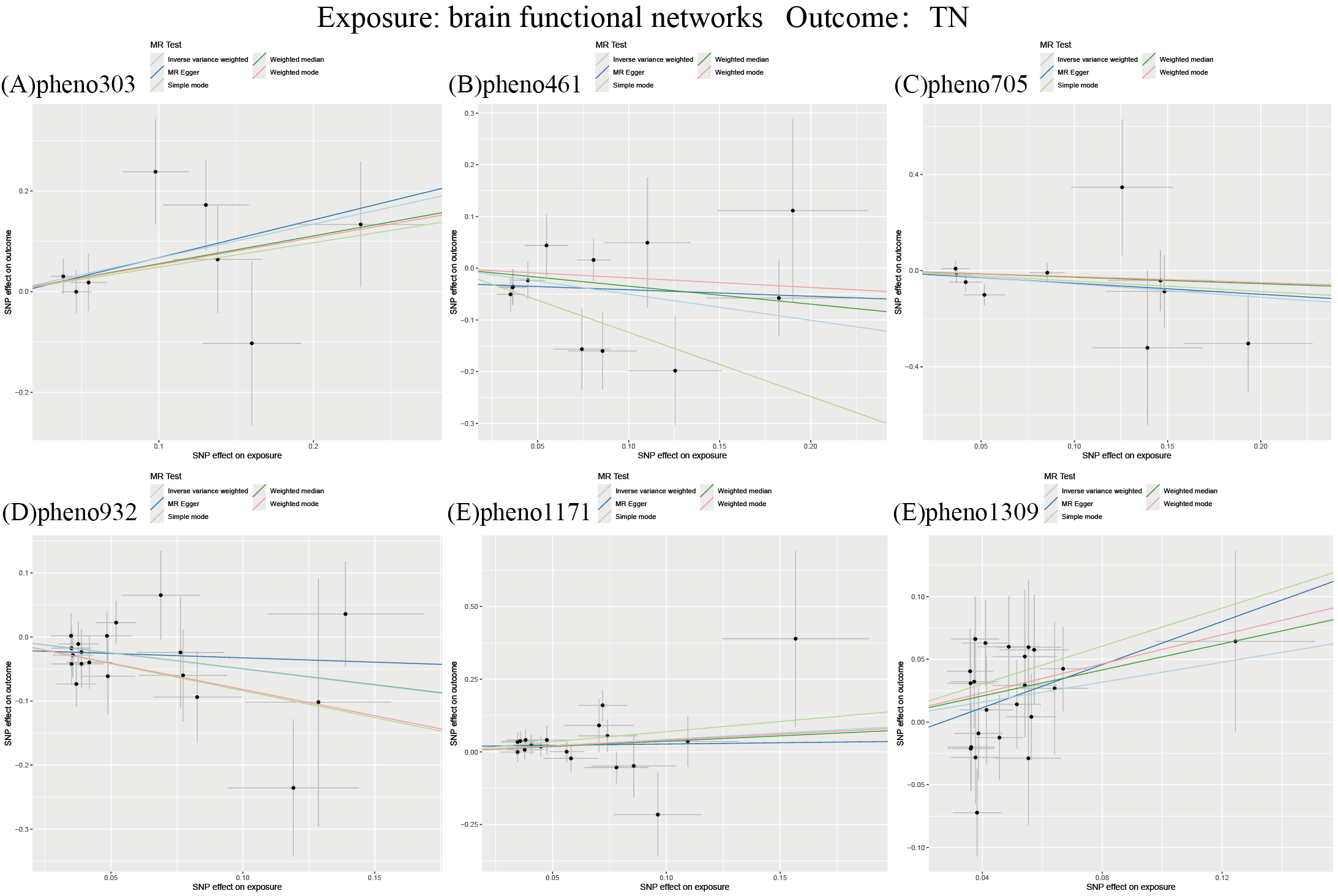


1. Scatter plots for "pheno303" on "TN"
2. Scatter plots "pheno461" on "TN"
3. Scatter plots "pheno705" on "TN"
4. Scatter plots for "pheno932" on "TN"
5. Scatter plots for "pheno1171" on "TN"
6. Scatter plots for "pheno1309" on "T

**Figure S7.** Forest plots for the effect of brain functional networks on TN.


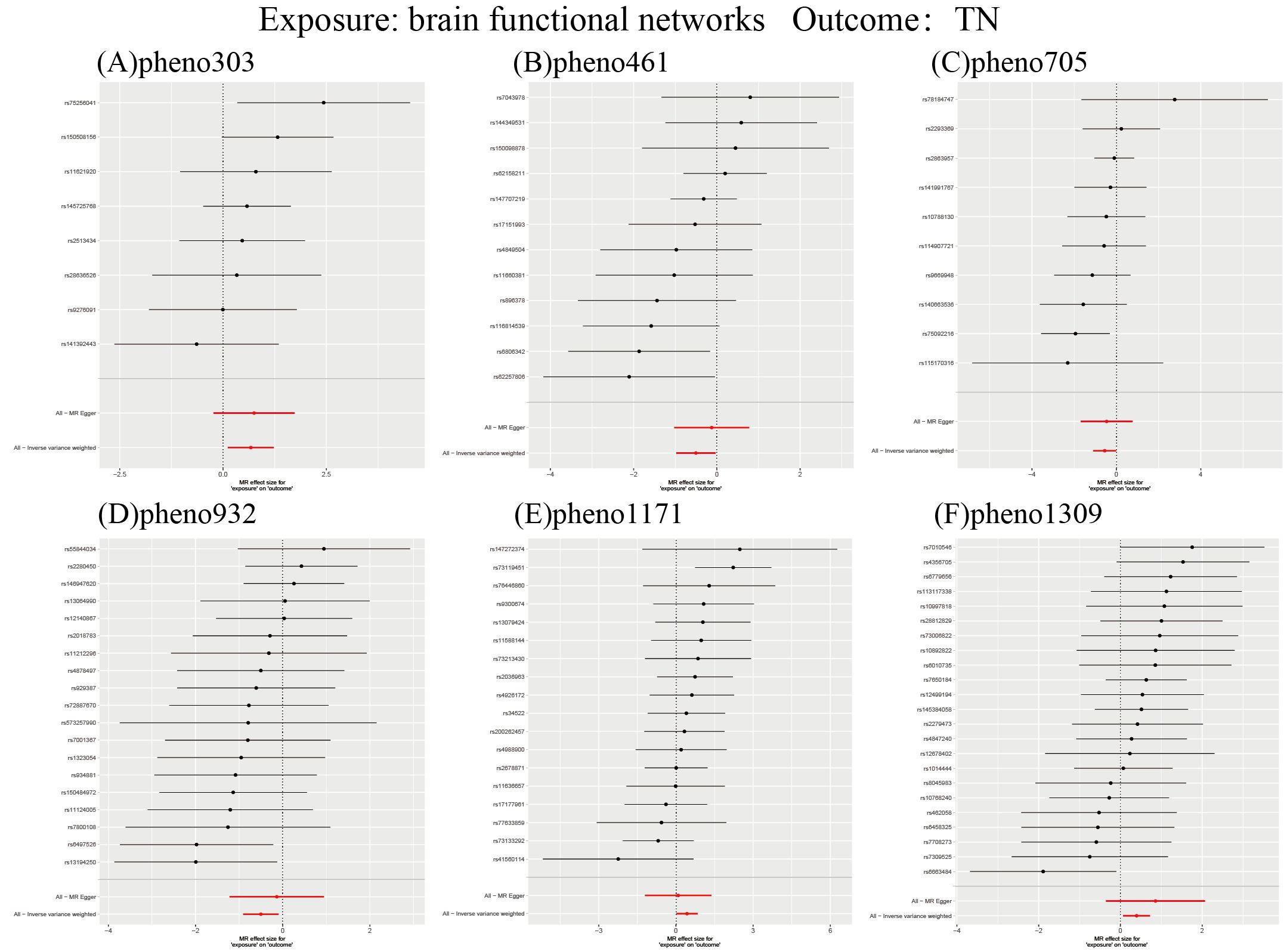


1. MR effect size for "pheno303" on "TN"
2. MR effect size for "pheno461" on "TN"
3. MR effect size for "pheno705" on "TN"
4. MR effect size for "pheno932" on "TN"
5. MR effect size for "pheno1171" on "TN"
6. MR effect size for "pheno1309" on "TN

**Figure S8.** Scatter plots,MR leave-one-out sensitivity analysis and Forest plots for the effect of inflammatory proteins on PHN and TN.


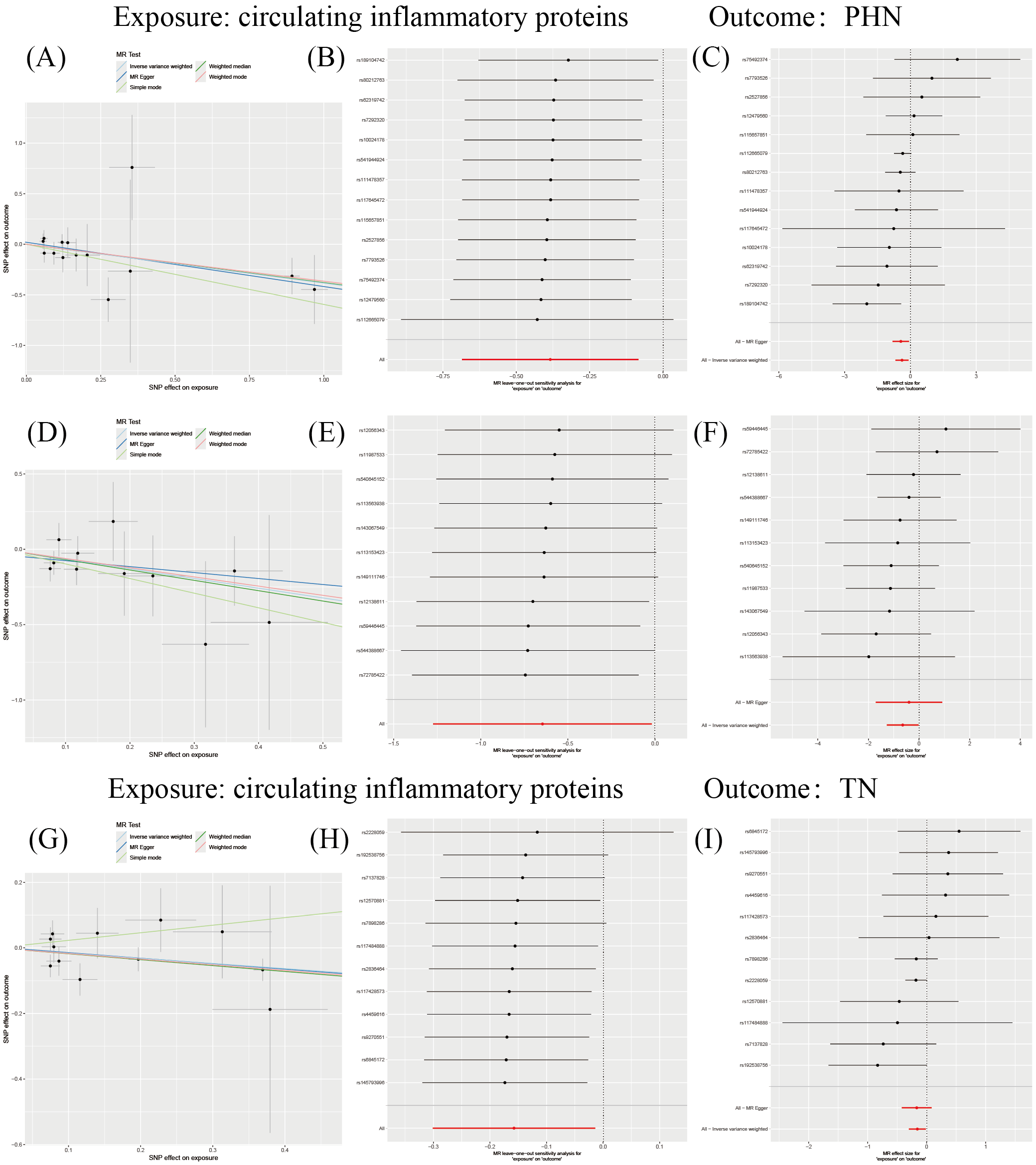


1. Scatter plots for "GCST90274759 ADA" on "PHN"
2. Analysis for "GCST90274759 ADA" on "PHN"
3. MR effect size for "GCST90274759 ADA" on "PHN"
4. Scatter plots for "GCST90274808 IL20RA" on "PHN"
5. Analysis for "GCST90274808 IL20RA" on "PHN"
6. MR effect size for "GCST90274808 IL20RA" on "PHN"
7. Scatter plots for "GCST90274800 IL15RA" on "TN"
8. Analysis for "GCST90274800 IL15RA" on "TN"
9. MR effect size for "GCST90274800 IL15RA" on "T

**Figure S9**. Scatter plots,MR leave-one-out sensitivity analysis and Forest plots for the effect of brain functional network ( Phneo12 ) on inflammatory protein ( IL20RA ).


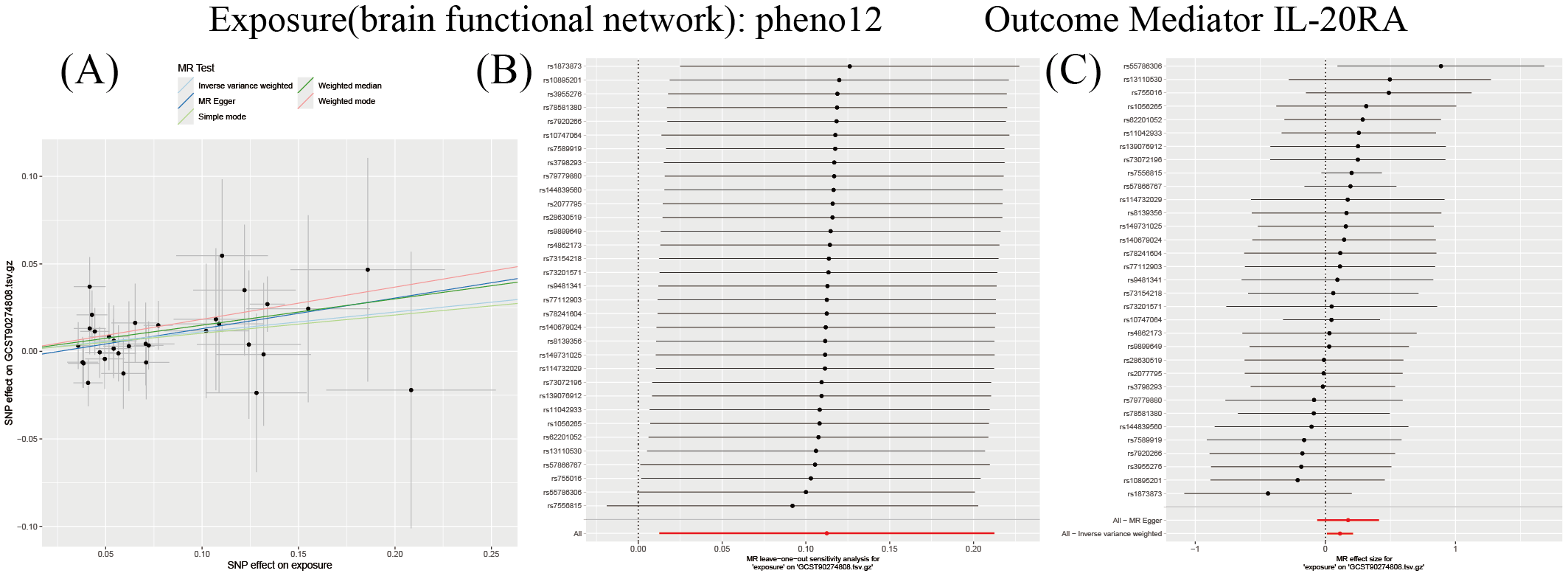


1. Scatter plots for "pheno12" on "IL20RA"
2. Analysis for "pheno12" on "IL20RA"
3. MR effect size for "pheno12" on "IL20RA"
